# Loss of endothelial EMCN drives tumor lung metastasis through the premetastatic niche

**DOI:** 10.1186/s12967-022-03649-4

**Published:** 2022-10-02

**Authors:** Guoxin Zhang, Mengyuan Li, Dandan Zhou, Xingjiu Yang, Wenlong Zhang, Ran Gao

**Affiliations:** 1 National Human Diseases Animal Model Resource Center, The Institute of Laboratory Animal Science, Chinese Academy of Medical Sciences & Peking Union Medical College, Beijing, 100021 China; 2 NHC Key Laboratory of Human Disease Comparative Medicine, Beijing Engineering Research Center for Experimental Animal Models of Human Critical Diseases, Beijing, China; 3 Beijing Engineering Research Center for Experimental Animal Models of Human Critical Diseases, Beijing, China

**Keywords:** EMCN, Lung metastasis, Host microenvironment, Neutrophil polarization, Endothelial cell

## Abstract

**Background:**

Metastasis is the primary cause of cancer-related mortality. Metastasis involves a complex multistep process during which individual tumor cells spread primarily through destruction of the endothelial barrier, entering the circulatory system to colonize distant organs. However, the role of the endothelial barrier as the rate-limiting process in tumor metastasis and how these processes affect the regulation of the host microenvironment at the molecular level are poorly understood.

**Methods:**

Here, we analyzed differentially expressed genes in breast cancer and lung adenocarcinoma, including metastatic and recurrent specimens, using TCGA dataset. The effects of EMCN on endothelial cells in vitro and in vivo were analyzed by assessing angiogenesis and vascular permeability, respectively. We established a syngeneic mouse model of endothelial cell-specific knockout of EMCN (EMCN^ecko^) to study the role of EMCN in tumor growth and metastasis. Transcriptome sequencing, Western blotting, qPCR and immunofluorescence confirmed important factors in the premetastatic niche. A mouse model of allograft tumor resection with lung metastasis was established to confirm the therapeutic effect of a notch inhibitor combined with an anti-TGF-β antibody.

**Results:**

We found a strong correlation of EMCN deficiency with tumor recurrence and metastasis. Comparative experiments in WT and EMCN^ecko^ mice revealed that endothelial EMCN deficiency did not affect primary tumor growth significantly but strongly promoted spontaneous metastasis. EMCN deficiency was associated with gene profiles that regulate cell junctions in vitro and enhance vascular permeability in vivo. Mechanistically, EMCN deficiency mainly affected the host microenvironment and led to the formation of a lung premetastatic niche by recruiting Ly6G^+^ neutrophils and upregulating MMP9, S100A8/A9 and TGF-β expression. Anti-TGF-β antibody effectively eliminated TGF-β-induced neutrophil polarization, thereby reducing lung metastasis. Notably, the combination of a Notch inhibitor and an anti-TGF-β antibody effectively inhibited tumor growth and lung metastasis and prolonged the survival time of mice.

**Conclusions:**

We present a new translational strategy of EMCN as a new key player in tumor lung metastasis by affecting the host microenvironment. These findings could provide a sound theoretical basis for clinical treatment.

**Supplementary Information:**

The online version contains supplementary material available at 10.1186/s12967-022-03649-4.

## Introduction

Metastasis of primary malignant tumors is the main cause of cancer-related mortality [1]. Only a few effective treatment options are available for patients with cancer metastasis [2]. Metastasis is a gradual process that includes the invasion and dissemination of malignant cells, colonization by circulating tumor cells, formation of a premetastatic niche and adaptation to the microenvironment of the metastatic site. Growing evidence shows that tumor metastasis colonization depends not only on abnormal gene changes in cancer cells but also on the premetastatic niche. Disseminated cancer cells successfully colonize distant organs by altering the local microenvironment to survive [3]. Therefore, interventions targeting the premetastatic niche may represent a new strategy for inhibiting tumor metastasis and therapeutic intervention for patients with metastatic cancers.

Neutrophils are the most abundant leukocytes (50–70%) in human blood circulation. Early studies have shown that neutrophils play a key role in inflammation and host resistance to microbial infections [4]. Growing evidence shows that neutrophils also play a significant role in tumor progression. However, neutrophils have been shown to possess both protumor and antitumor properties, but this finding is controversial. Lev Becker et al. found that the neutrophil-derived antitumor molecule ELANE can selectively kill tumor cells and attenuate tumorigenesis [5]. It has also been found that the interaction between neutrophils and circulating tumor cells in blood promotes the cell cycle progression and the metastatic potential of circulating tumor cells [6]. The inflammatory factors released by neutrophils stimulated by ovarian tumors become neutrophil extracellular traps and promote the formation of a premetastatic niche and ovarian cancer cell metastasis [7].

Endothelial cells form blood vessels, support tumor growth by providing nutrition and oxygen, and play an important role in cancer metastasis [8]. These cells provide vascular secretory factors to coordinate tumor progression. Constitutive activation of vascular Notch signaling promotes metastasis by activating proinflammatory and senescence signaling in endothelial cells [9]. EMCN is a transmembrane *O*-sialylated protein expressed on the surface of the endothelium. Human and mouse EMCN contain 261 amino acid residues and possess an extracellular domain rich in serine and threonine residues [10, 11]. Functionally, EMCN has been reported to affect tube morphogenesis of endothelial cells in vitro and leukocyte adhesion to endothelial cells in the blood [12, 13]. EMCN/MUC15 combined analysis has been suggested as a prognostic signature of gastric cancer [14]. Endothelial Notch activation downregulates EMCN and promotes the cross endothelial migration of neutrophils in vitro*,* thus modulating acute inflammation in hepatic ischemia/reperfusion injury [15].

Herein, we report a new mechanism by which EMCN deficiency leads to tumor metastasis independent of tumor growth. EMCN deficiency is associated with gene profiles related to the regulation of cell junctions in vitro and vascular permeability in vivo. Furthermore, we demonstrate that EMCN deficiency leads to the formation of a premetastatic niche to promote tumor metastasis in a neutrophil-dependent manner. A Notch inhibitor combined with an anti-TGF-β antibody attenuated tumor metastasis. Of note, TCGA data showed that patients with high EMCN or low Notch1 expression survived longer than those with low EMCN or high Notch1 expression, indicating that EMCN and Notch levels have therapeutic and prognostic potential.

## Materials and methods

### Animal models

EMCN^flox/flox^ mice and Tek-CreERT2 mice were purchased from Beijing VIEWSOLID Biotechnology Co., Ltd., and raised in a temperature-controlled facility with a 12-h light/dark cycle in a specific pathogen-free environment in a separate ventilation cage. Mice had ad libitum access to food and water. All animal experiments were approved by the Animal Experiments Committee of the Chinese Academy of Medical Sciences (IACUC: GR21003). EMCN^flox/flox^ mice were crossed with Tek-CreERT2 mice to generate endothelial cell-specific EMCN knockout mice (EMCN^ecko^). All female or male mice aged 8–10 weeks (weight 23–25 g) were randomly divided into groups for follow-up experiments. All genotyping was confirmed by PCR. Gene deletion by Cre recombinase was achieved by intraperitoneal (i.p.) injection of tamoxifen (75 mg/kg body weight) (Sigma‒Aldrich, CAS# 10540-29-1) every day for 5 days, starting seven days before tumor cell injection. EMCN knockout efficiency was determined by Western blot.

### Cell culture, proliferation and angiogenesis

LLC (LL/2) murine lung carcinoma cells, B16-F10 melanoma cells and PUMC-HUVEC-T1 (later HUVEC, SV40T transformed immortalized human umbilical vein endothelial cells) were purchased from the National Infrastructure of Cell Line Resource and maintained in DMEM (Gibco) supplemented with 10% FBS (Gibco). The human salivary gland adenoid cystic carcinoma cell line SACC-LM (highly invasive) was obtained from the Peking University Hospital of Stomatology. The cells were cultured in RPMI 1640 supplemented with 10% fetal bovine serum (FBS) and a 1% penicillin‒streptomycin solution (Invitrogen). Cell line authentication was performed by short tandem repeat (STR) assessment. All experiments were performed with mycoplasma-free LLC-luciferase clones produced by continuous puromycin (800 μg/ml) screening of LLC cells overexpressing the lentiviral-driven luciferase gene. For EMCN deletion, three human shRNA sequences were cloned into a plasmid vector, and lentivirus was packaged by Shanghai Jikai GENE Biological Company. HUVECs were infected with lentivirus (MOI = 20). HUVEC cell lines with stable EMCN knockdown were screened by puromycin. Negative control and EMCN knockdown were termed HUVEC/shNC and HUVEC/shEMCN, respectively. HUVEC proliferation was analyzed by CCK8 kits (Dojindo, C0038). For angiogenesis, the concentrated and reduced growth factor matrix gel (Corning) was placed into a 24-well plate, and the plate was incubated at 37 °C for 30 min. HUVECs/shNC and HUVECs/shEMCN (1 × 10^5^ cells) suspended in serum-containing media were added to 24-well plates with solidified Matrigel. Images were obtained with a contrast microscope and analyzed using the Fiji plug-in for ImageJ software.

### Cell infection and generation of conditioned medium

HUVECs were infected with lentivirus (MOI = 20). Cells with stable NICD knockdown were selected in puromycin-supplemented medium. NICD knockout efficiency and EMCN expression were detected by Western blotting. Condition medium (CM) was prepared by seeding 1 × 10^6^ LLC, SACC-LM and B16-F10 cells in common culture medium supplemented with 10% FBS for 48 h. The harvested CM was then centrifuged at 250*g* for 10 min to remove cells and debris. Then, HUVECs were washed with PBS and incubated further for 48 h in different tumor cell-conditioned media.

### Syngeneic LLC tumor model, metastasis and drug administration

#### Subcutaneous syngeneic mouse tumor model

Syngeneic LLC or B16-F10 cells (5 × 10^5^ suspended in PBS) were injected s.c. into the indicated mice (female or male mice aged 8–10 weeks). Fourteen days later, the tumors were harvested and analyzed further. Tumor formation and body weight were monitored every other day. The tumor volume was calculated as V = L × W^2^/2, where L and W are the length and width of the tumor, respectively.

#### Intravenous injection of lung metastasis mouse model

One week after tamoxifen-induced EMCN knockout, luciferase reporter LLC or wild-type (1 × 10^6^ suspended in PBS) tumor cells were injected into the mouse tail vein. Mice were randomly assigned to different experimental groups. After 21 days of tumor cell injection, the lung was dissected to observe the metastatic foci, and the tissue was fixed for subsequent section staining. For the neutrophil deletion experiment, mice were administered either anti-Ly6G^+^ antibody (clone 1A8; Bio X Cell, 7.5 mg/kg) or control (PBS) once every three days in vivo.

#### Postsurgical metastasis model

Syngeneic LLC tumor cells (3 × 10^5^ or 5 × 10^5^ suspended in PBS) were subcutaneously injected into the flanks of EMCN^ecko^ and control mice 1 week after tamoxifen administration. Primary tumor growth was measured, and tumor growth was calculated. The primary tumor (approximately 1 cm in diameter) was surgically removed 15 or 21 days after implantation under anesthesia. The tissue was fixed for subsequent section staining, and the tumor was weighed. We observed lung metastasis 1 week, 2 weeks and 3 weeks after tumor resection by HE staining. After 21 days of primary tumor resection, LLC-injected mice showed significant distant lung metastasis. Therefore, 21 days after resection was selected as the end point of our follow-up experiments. For the therapy experiment, mice were administered either DAPT (GSI-IX, 20 mg/kg) or vehicle (DMSO:PEG400:Tween-80:NaCl = 10%:40%:5%:45%) once a day. The primary tumor was surgically removed with a diameter of 1 cm until the experimental end point criteria were reached. DAPT or vehicle was injected once every three days. For the combination treatment experiment, mice were administered either DAPT or vehicle once a day. Anti-TGF-β antibody (1D11; Bio X Cell 10 mg/kg) was injected once every three days. The primary tumor was surgically removed when a diameter of 1 cm was obtained or when the experimental endpoint criteria were reached. The investigators assessing endpoint criteria were blinded to the treatment administered. For survival experiments, humane endpoints included weight loss of 20% or greater, reduced activity, pale feet and visible symptoms of distress, such as hunching, closed eyes and isolation from cage mates.

### Depletion of neutrophils and in vivo imaging system (IVIS)

To deplete Ly6G^+^ neutrophils, mice were intraperitoneally injected with 7.5 mg/kg anti-Ly6G^+^ (clone 1A8; BioXCell) once every three days. Neutrophil depletion was confirmed by immunofluorescence. For visualization of the luciferase reporter gene that is expressed by the LLC cells, d-Luciferin (PerkinElmer cat. #122796) was intraperitoneally injected (150 mg/kg), and the lungs were dissected 10 min after injection. Luciferase-positive regions were imaged using IVIS Lumina II (Caliper Life Sciences).

### In vivo vessel permeability assay

Tumor-bearing WT mice and EMCN^ecko^ mice were injected intravenously with 100 µl of a mixture containing 2 mg/ml rhodamine-conjugated dextran (70 kDa) in PBS. After 30 min, the mice were euthanized, and cardiac perfusion was performed with 10 ml PBS. Lung tissue was fixed with 4% paraformaldehyde (PFA) at 4 °C for 24 h; washed in PBS for 5 min; passed through sucrose solutions of 10%, 20% and 30% for 24 h; and embedded in OCT. Frozen blocks were cut into 10-µm cryosections. Images were obtained using a Leica confocal microscope. The fluorescence intensity of 70 kDa rhodamine-dextran was quantified by using ImageJ software.

### Immunofluorescence (IF) and immunohistochemistry (IHC)

Lung and tumor tissues were fixed in 10% formaldehyde solution in PBS at room temperature followed by routine dehydration, paraffin embedding, and tissue sectioning. Paraffin sections (4 µm) were stained with hematoxylin–eosin to observe the structure of the main organs and lung metastasis. For immunofluorescence staining, in brief, paraffin sections were dewaxed and placed into water. The sections were placed into sodium citrate buffer, heated in a microwave oven for antigen repair, restored to room temperature, and blocked with goat serum at room temperature for 1 h. Sections were incubated with the primary antibodies anti-Ly6G^+^ (ab238132, 1:200), anti-Arg2^+^ (ab264071, 1:200) and anti-NOS2 (ab115819, 1:200) at 4 ℃ overnight. The slices were washed thrice with PBS for 3 min each time followed by incubation with Alexa Fluor 488- or 594-conjugated secondary antibodies (1:5000 dilutions) for 1 h. DAPI was used to stain the nucleus for 15 min, and samples were subject to imaging with a Leica fluorescence microscope. A routine protocol was performed for immunohistochemistry. The primary antibody dilutions were S100A8/A9 (ab22506, 1:1000), anti-CD31 (D8V9E, 1:500), and anti-Ki67 (ab16667, 1:1000).

### Quantitative real-time PCR

Total RNA from cultured cells (HUVECs/shNCs and HUVECs/shEMCNs) and mouse lung tissues was isolated with TRIzol reagent (Invitrogen) as instructed. cDNA was synthesized from 2 μg of total RNA with random primers using a Thermo kit, and the concentration was measured by Colibri. mRNA expression was assessed based on the threshold cycle (Ct), and relative expression levels were calculated as 2^−ΔΔct^ after normalization to GAPDH expression. The primers used for quantitative real-time PCR are listed in Additional file 6: Table S1.

### Western blotting

Western blot analysis was implemented using a standard protocol. The primary antibodies used for Western blot analysis included anti-endomucin (sc-65495, 1:500), anti-endomucin (ab96315, 1:1000), anti-claudin-5 (ab131259, 1:1000), anti-MMP-9 (ab283575, 1:1000), anti-ZO-1 (ab216880, 1:1000), and anti-TGF-β (ab215715, 1:1000). The secondary antibodies included goat anti-mouse (ZB-2055, 1:10000), goat anti-rat (ZB-2040, 1:10000) and goat anti-rabbit (ZB-2306, 1:10000). Anti-β-actin (ab8226, 1:2000) was used as a loading control.

### RNA isolation and library preparation

Total RNA was extracted using TRIzol reagent according to the manufacturer’s protocol. RNA purity and quantification were evaluated using a NanoDrop 2000 spectrophotometer (Thermo Scientific, USA). RNA integrity was assessed using the Agilent 2100 Bioanalyzer (Agilent Technologies, Santa Clara, CA, USA). Then, the libraries were constructed using the TruSeq Stranded mRNA LT Sample Prep Kit (Illumina, San Diego, CA, USA) according to the manufacturer’s instructions. Transcriptome sequencing and analysis were performed by OE Biotech Co., Ltd. (Shanghai, China).

### RNA sequencing and differentially expressed gene analysis

The libraries were sequenced on an Illumina HiSeq X Ten platform, and 150-bp paired-end reads were generated. Transcriptome sequencing and analysis were performed by OE Biotech (Shanghai, China). Raw data (raw reads) were processed using Trimmomatic [16]. The reads containing poly-*N* and the low-quality reads were removed to obtain clean reads. Then, the clean reads were mapped to the reference genome using HISAT2 [17]. The FPKM [18] value of each gene was calculated using cufflinks [19], and the read counts of each gene were obtained by htseq-count [20]. DEGs were identified using the DESeq R package functions estimate SizeFactors and nbinomTest. A P value < 0.05 and fold change > 2 or fold change < 0.5 were set as the thresholds for significant differential expression. Hierarchical cluster analysis of DEGs was performed to explore gene expression patterns. GO enrichment and KEGG [21] pathway enrichment analyses of DEGs were performed using the R package based on the hypergeometric distribution. The sequencing coverage and quality statistics for each sample are summarized in Additional file 7: Table S2.

### TCGA database

TCGA database provides high-throughput analysis of different tumors, including data on mRNA expression. Combining bioinformatics analysis with patient clinical information lays a foundation for improving cancer prevention and discovering new targets for treatment. In this study, we downloaded RNA-seq data of lung adenocarcinoma from TCGA database. mRNA data from 594 samples, including 59 normal samples and 535 lung cancer samples, and mRNA data from 1222 samples, including 112 normal breast samples and 1110 breast cancer samples, were used in this analysis. Using the R software package, the downloaded data were normalized and differentially analyzed to obtain differentially expressed mRNAs. We used the R package Survival to examine the prognostic potential of EMCN expression levels in cancers. Survival-relevant mRNAs with a log-rank P value < 0.05 were considered significant. The relevant data provided by TCGA are publicly available.

### Statistical analysis

All graphs and statistical analyses were completed using GraphPad Prism software v8.0. Significant differences were evaluated using an independent sample T test, and multiple treatment groups were compared within individual experiments by ANOVA. TCGA data were downloaded from the cBioPortal website, and the log-rank test was used for comparison of survival outcomes with the Kaplan‒Meier method. Values of p < 0.05 were considered significant. All values are presented as the mean ± SD.

## Results

### Downregulation of EMCN expression is related to metastasis and recurrence in tumor patients

**Fig. 1:**
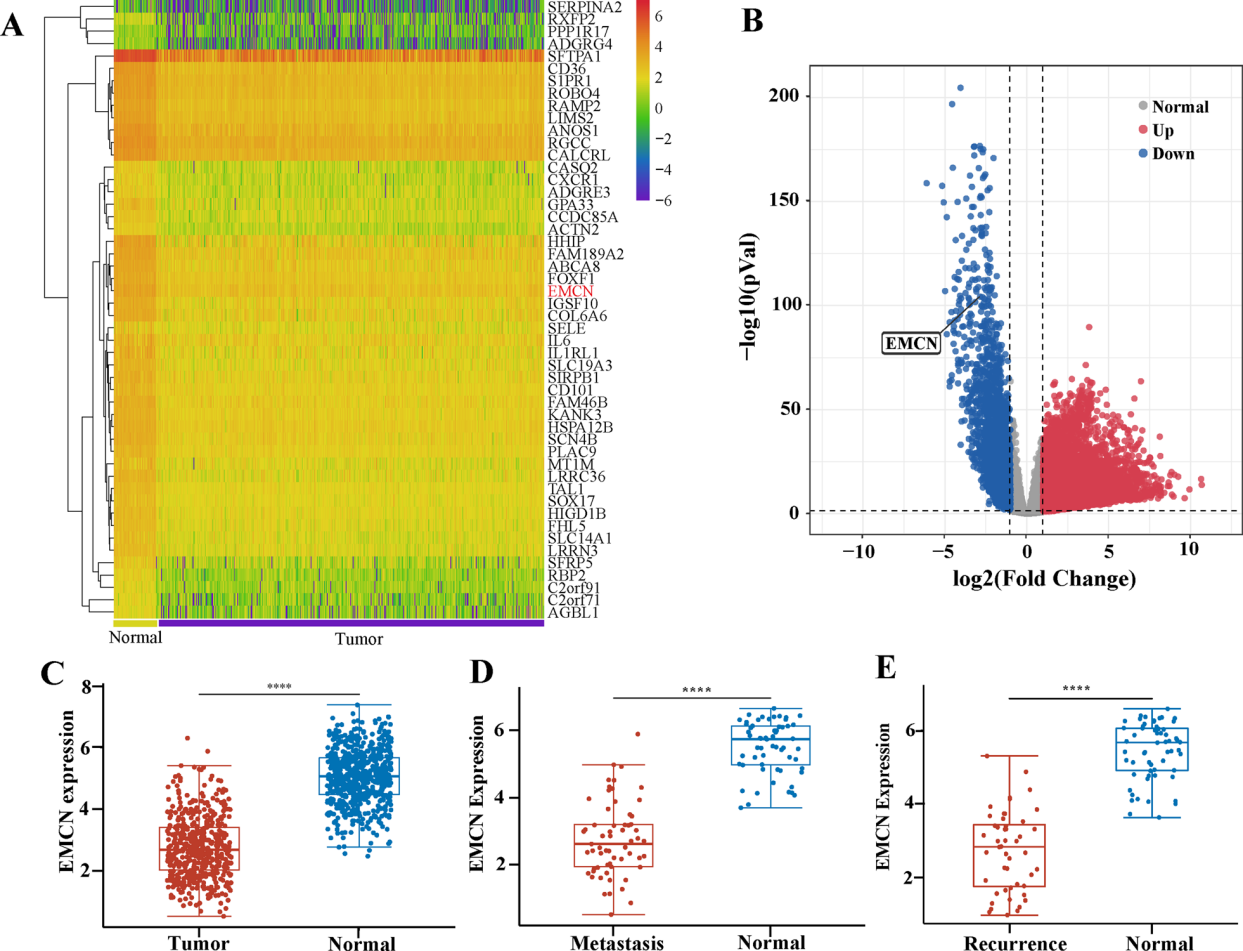
The downregulation of EMCN expression is related to metastasis and recurrence in lung cancer patients. **A** Heatmap of differentially expressed genes in lung cancer from TCGA datasets. **B** Volcano plot of differentially expressed mRNAs. Red dots represent upregulated mRNAs, and blue dots represent downregulated mRNAs. **C** EMCN expression levels in adjacent tissues and tumor tissues were analyzed (Wilcox tests, ****p < 0.0001, **p < 0.01, **p < 0.05). **D** EMCN gene expression levels in metastatic patients and normal tissues in the TCGA dataset (Wilcox tests, ****p < 0.0001). **E** EMCN gene expression levels in recurrence patients and normal tissues in TCGA dataset (Wilcox tests, ****p < 0.0001)

To identify the key genes and pathways involved in tumor metastasis, we compared the transcriptomic profiles of 55 normal and 538 lung cancer samples from TCGA database. In total, 3719 genes were significantly upregulated, and 1811 genes were significantly downregulated (Fig. 1A and B). Similar transcriptomic analysis of 112 normal 1110 breast cancer samples revealed upregulation of 7393 and downregulation of 3199 genes (Additional file 1: Fig S1A and S1B). Given that tumor cell interactions with endothelial cells play a critical role in cancer metastasis [22], we first focused on the expression analysis of these genes. We found that EMCN is specifically expressed in microvascular endothelial cells and showed remarkable downregulation in the majority of both metastatic lung and breast tumors. We hypothesized that EMCN downregulation may be important for tumor metastasis and recurrence (Fig. 1C and Additional file 1: Fig. S1C). To further investigate the clinical significance of EMCN expression in metastatic lung and breast cancer patients, we analyzed EMCN expression in patients with and without metastasis. The results showed that EMCN expression was significantly reduced in metastatic tumors (Fig. 1D and Additional file 1: Fig. S1D) and was strongly reduced in tumor recurrence (Fig. 1E and Additional file 1: Fig. S1E). These analyses revealed an important role of EMCN in the progression of tumor metastasis.

### EMCN expression in endothelial cells does not affect tumor growth in vivo

**Fig. 2:**
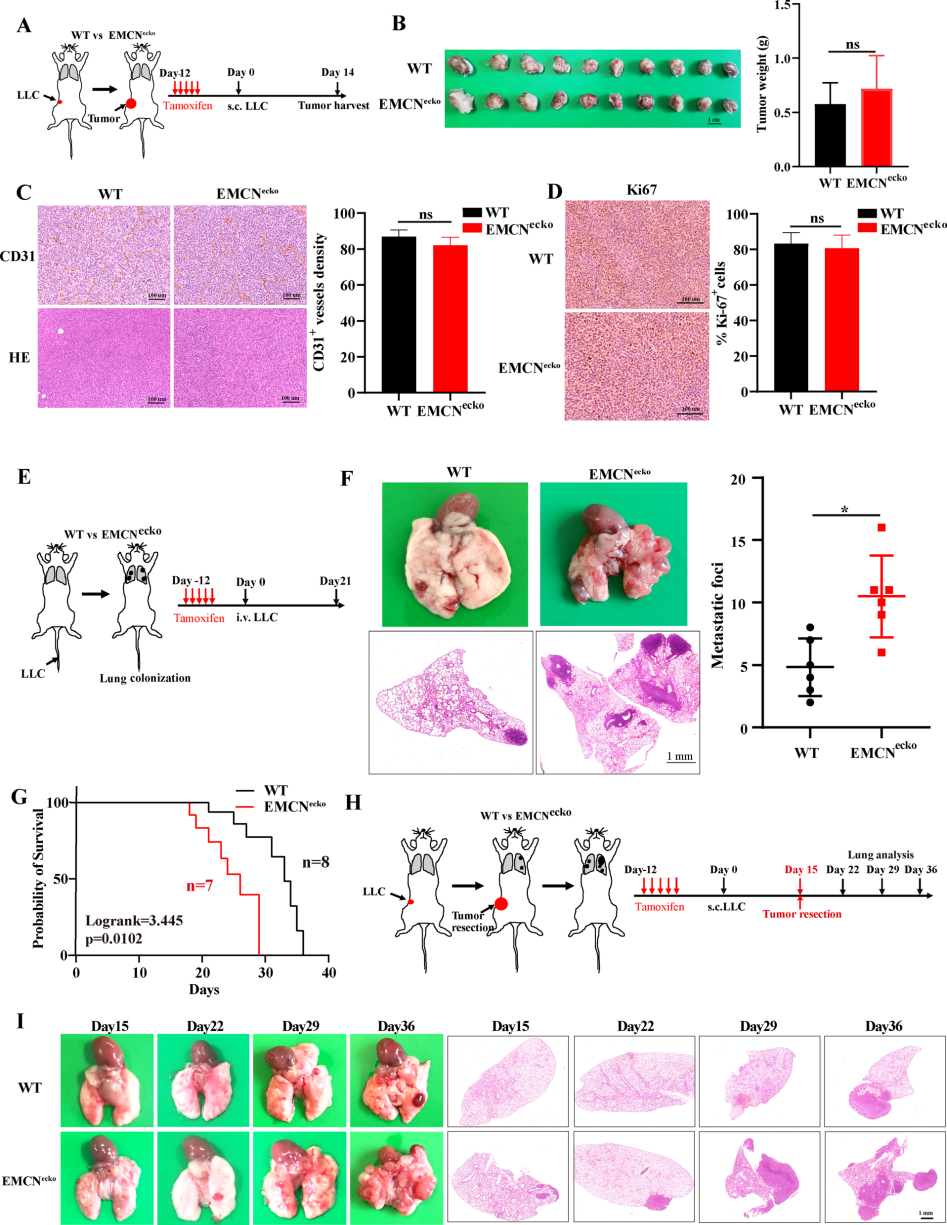
EMCN in ECs does not affect tumor growth or angiogenesis and aggravates spontaneous lung metastasis from subcutaneous syngeneic tumors. **A** The schematic diagram shows the experimental design of tamoxifen induction, subcutaneous implantation of LLC tumor cells and tumor harvest. **B** LLC cells were subcutaneously injected into WT and EMCN^ecko^ mice for 14 days. LLC tumors were dissected and photographed (left). Tumor weight was measured by a precision balance (right, data represent the mean ± SD, n = 10 mice per group). Statistical analysis was performed using unpaired Student’s t test. **p < 0.01. **C** Representative CD31 staining and HE images of tumor sections from WT and EMCN^ecko^ mice (left panel) are shown. CD31 indicates vascular density. Quantification of CD31 expression was performed (right panel). (Data represent the mean ± SD, n = 4). Statistical analysis was performed using unpaired Student’s t test. ns: not significant. Scale bars: 100 μm. **D** Representative Ki67 staining images of tumor sections from WT and EMCN^ecko^ mice are shown (left panel). Quantification of Ki67 expression was performed (right panel) (data represent the mean ± SD, n = 4). Statistical analysis was performed using unpaired Student’s t test. Ns, not significant. Scale bars, 100 μm. **E** The schematic diagram shows the experimental design of tamoxifen induction, intravenous injection of LLC tumor cells and pulmonary analysis. **F** Representative images of lungs harvested from WT and EMCN^ecko^ mice after 21 days of LLC tumor implantation (left, n = 6). Metastatic foci were quantified (right, data represent the mean ± SD, n = 6). Statistical analysis was performed using unpaired Student’s t test. *p < 0.05. **G** Overall survival of intravenous pulmonary metastasis in WT and EMCN^ecko^ mice. EMCN^ecko^ (n = 7), WT (n = 8). The EMCN^ecko^ mouse groups showed worse overall survival (p = 0.0102). Statistical analysis was performed using the log-rank test. **H** The schematic diagram shows the experimental design of tamoxifen induction, subcutaneous implantation of LLC tumor cells, tumor resection and lung analysis at different time points. **I** Representative HE-stained images of lung sections from WT and EMCN^ecko^ tumor-bearing mice at different time points after surgery. The left panel shows lung metastasis images, and the right panel shows HE-stained sections

We used an inducible knockout model using Tek-creERT2 mice to drive endothelial-specific deletion of EMCN (hereafter referred to as EMCN^ecko^). Endothelial EMCN inactivation was confirmed at the gene level by PCR and at the protein level by Western blot (Additional file 2: Fig. S2A and S2B). EMCN^ecko^ mice appeared phenotypically normal; however, histological examination revealed an increase in red blood cells in the lungs of these animals compared with the wild-type (WT) mice (Additional file 2: Fig. S2C). To determine whether EMCN mediates tumor growth, syngeneic LLC cells were injected subcutaneously into the flanks of WT and EMCN^ecko^ mice. Neoplasms were formed within 2 weeks and displayed no distinct impairment in growth rate or tumor angiogenesis between EMCN^ecko^ and WT mice, which was also evidenced by Ki67 and CD31 staining, respectively (Fig. 2A–D). Consequently, EMCN expression in ECs does not affect tumor growth in vivo.

### EMCN deficiency in ECs aggravates spontaneous lung metastasis.

Cancer cells penetrate blood vessels through intravasation and extravasation, and both processes require the ability to break through endothelial cells [23]. Tumor cell metastasis typically occurs in small capillaries [24], and EMCN is a glycoprotein expressed in endothelial cells of these capillaries. We hypothesized that EMCN depletion in tumors may obliterate the integrity of the endothelial barrier and lead to an increase in cell infiltration. To determine the effect of EMCN deficiency on tumor metastasis in vivo (Fig. 2E), EMCN^ecko^ mice were injected with LLC cells through the tail vein. The pathology results showed significantly higher metastasis in EMCN^ecko^ mice (Fig. 2F), suggesting that EMCN depletion caused an increase in metastasis. Furthermore, EMCN knockout mice showed significantly reduced survival compared to WT mice when LLC cells were injected into the venous circulation (Fig. 2G).

To construct a mouse model that is more consistent with the metastasis of clinical tumor patients, we resected subcutaneous tumors 2 weeks after implantation to simulate surgical resection in tumor patients and allowed time for disseminated tumor cells to form detectable metastases. The effect of EMCN knockout on lung metastasis was observed after resection of LLC primary tumors (Fig. 2H). EMCN^ecko^ mice exhibited a significant increase in metastasis upon primary tumor resection compared with WT mice (Fig. 2I and Fig. S2D). We used a second animal model to confirm the same results, indicating that the occurrence of lung metastasis is not cell specific (Additional file 2: Fig. S2E). These results demonstrated that (i) loss of EMCN in the vascular endothelium results in markedly greater spontaneous metastases to the lung and (ii) the establishment of EMCN^ecko^ model mice with greater spontaneous metastases.

### EMCN deficiency affects gene expression profiles associated with cell junctions and vascular permeability

**Fig. 3:**
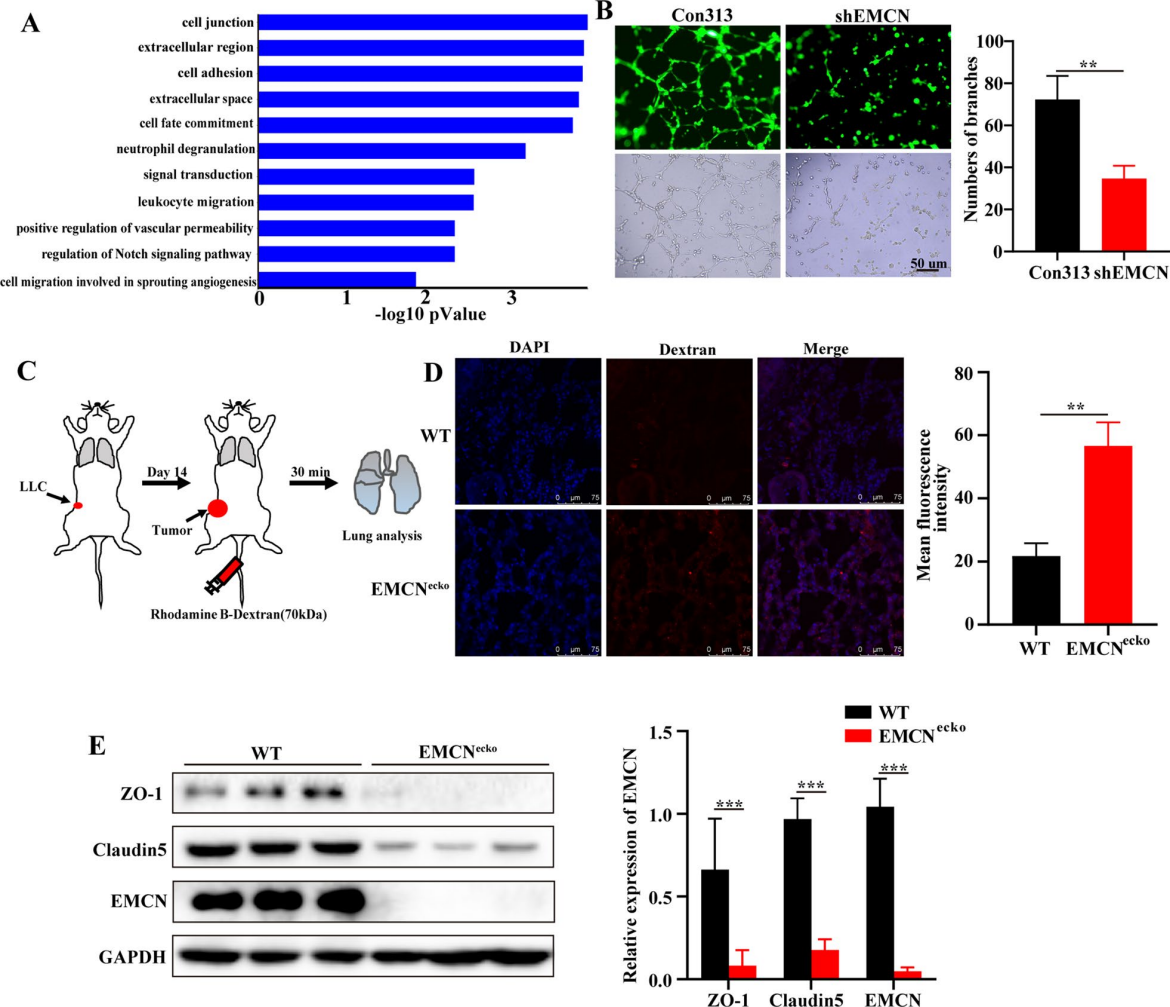
EMCN deficiency affects gene expression profiles linked to cell junction and vascular permeability. **A** Functional enrichment analysis of differentially expressed genes in HUVECs and HUVECs/shEMCN was performed using KEGG pathways. **B** Formation of capillary-like structures by HUVECs transduced with control-shRNA (Con313) or shEMCN and cultured on Matrigel matrix in 24-well plates (left). Quantification of tubular morphogenesis induced in HUVECs. Tube formation was determined by the numbers of branches (right). Data represent the mean ± SD. Statistical analysis was performed using unpaired Student’s t test. **p < 0.01. **C** Schematic representation of the rhodamine B-dextran lung extravasation assay procedure. **D** Representative images of lung sections show extravasation of rhodamine B-dextran (red) after intravenous injection of rhodamine B-dextran (left). Quantification of the mean fluorescence intensity of lung permeability in subcutaneous tumors in WT and EMCN^ecko^ mice (right). Data represent the mean ± SD. Statistical analysis was performed using unpaired Student’s t test. **p < 0.01. Scale bar, 75 μm. **E** Western blot analysis of the expression levels of ZO-1, Claudin5 and EMCN in lung samples from WT and EMCN^ecko^ tumor-bearing mice. The gray value was quantified by ImageJ. (Data represent the mean ± SD). Statistical analysis was performed using unpaired Student’s t test. ***p < 0.001

To further understand the mechanism of endothelial EMCN in tumor metastasis, we constructed a stable EMCN knockdown cell line in vitro. The results showed that EMCN knockdown significantly inhibited endothelial cell proliferation (Additional file 3: Fig. S3A and S3B). Next, we evaluated the gene expression profiles of EMCN (HUVEC/shEMCN) knockdown and control endothelial cells (HUVEC/Con313). Differentially expressed genes were selected as those showing either upregulation or downregulation by > twofold in HUVECs/shEMCN compared with HUVECs/Con313. KEGG analysis of differentially expressed genes showed that EMCN knockdown in HUVECs significantly affected cell junctions (Fig. 3A). Patricia A. D’Amore et al. reported that EMCN controls angiogenesis by regulating the activation of VEGFR2 in vitro [13]. We further confirmed whether EMCN controls endothelial cell junctions in vitro. HUVECs/Con313 and HUVECs/shEMCN were seeded on Matrigel to facilitate capillary tube formation. Strikingly, EMCN deficiency impeded HUVEC angiogenesis, which is consistent with previous studies [13] (Fig. 3B). In the in vivo study, we found that EMCN did not affect angiogenesis but did affect tumor metastasis. By comparing EMCN expression levels in naive, premetastatic and metastatic lung tissue, we found that EMCN expression levels were decreased significantly in tumor metastasis (Additional file 3: Fig. S3C). Thus, we hypothesized that endothelial EMCN deficiency changes vascular barrier properties, such as vascular permeability. Thus, we established a pulmonary microvascular permeability model by intravenous injection of rhodamine-dextran (70 kDa). After 30 min, the permeability of high-molecular-weight rhodamine in the lung parenchyma was analyzed by fluorescence microscopy. The results revealed slight leakage in the lungs of EMCN^ecko^ mice compared with WT mice (Additional file 3: Fig. S3D). Interestingly, EMCN^ecko^ tumor-bearing mice exhibited significantly increased lung vascular permeability compared with WT mice, as indicated by the fluorescence signal intensity observed in frozen lung sections (Fig. 3C, D). The intercellular junction of endothelial cells composed of adhesive junctions and tight junctions is the key factor in maintaining vascular integrity. ZO-1 and Claudin5 are important adhesion molecules that maintain endothelial cell‒cell contact [25–27]. To explore how EMCN regulates vascular permeability in vivo, we further detected changes in ZO-1 and Claudin5 in the lungs of WT and EMCN^ecko^ tumor-bearing mice. The results showed that EMCN deficiency caused a significant reduction in ZO-1 and Claudin5 expression (Fig. 3E). In brief, these results suggested that EMCN depletion affected the expression of ZO-1 and Claudin5 and the integrity of the vascular barrier of pulmonary capillaries, causing an increase in vascular permeability.

### EMCN deficiency leads to the formation of a lung premetastatic niche by upregulating MMP9, TGF-β and S100A8/A9

**Fig. 4:**
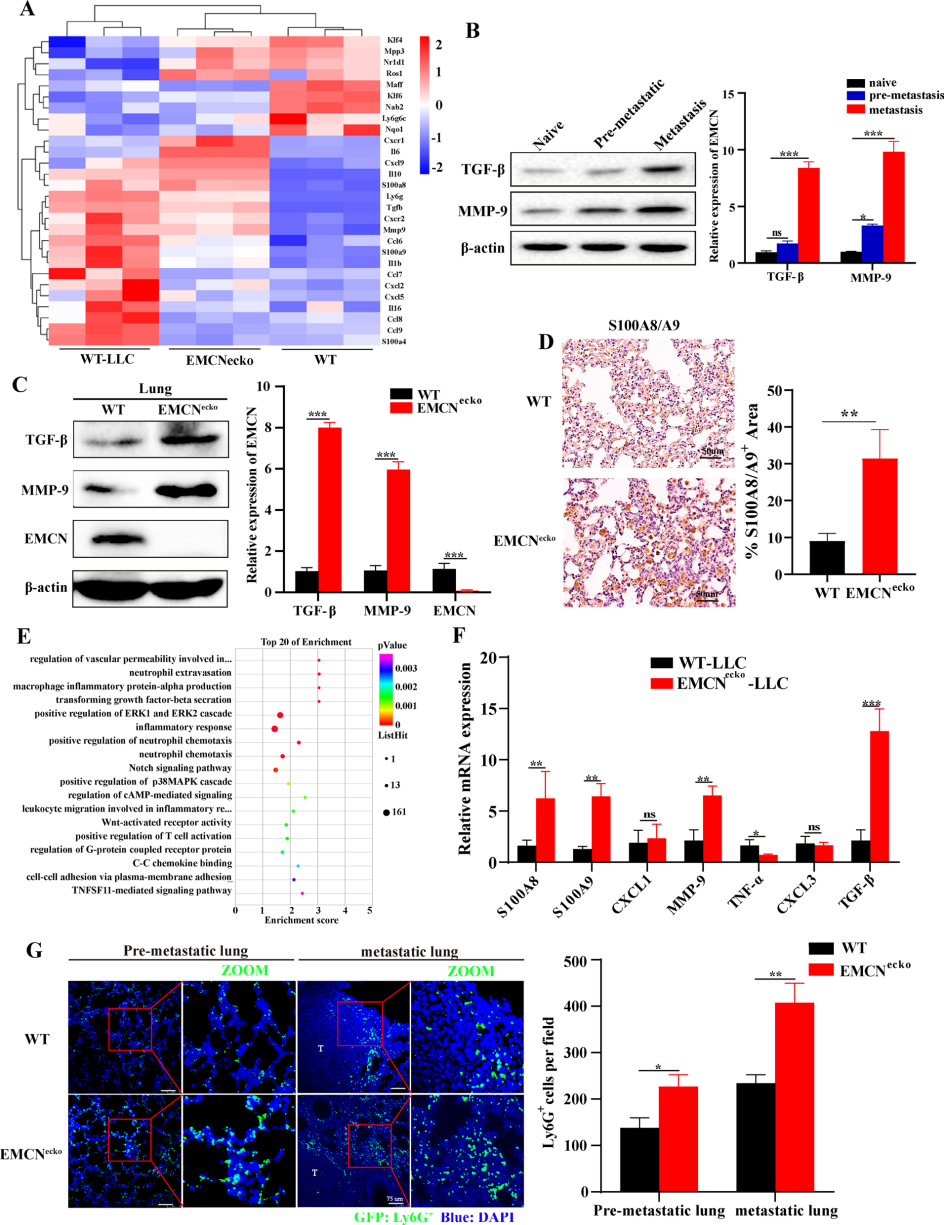
EMCN deficiency leads to the formation of a lung premetastatic niche. **A** Representative heatmap of differentially expressed genes in naive lung (WT), lung in the microenvironment before tumor metastasis (subcutaneous inoculation of tumor for 21 days) and EMCN^ecko^ mouse lung. Each row represents a gene, and each column represents a group of mice. **B** TGF-β and MMP-9 expression in naive, premetastatic and metastatic lungs. TGF-β and MMP-9 expression levels in naive, premetastatic and metastatic lungs were quantified by Western blotting. Each experiment was repeated thrice. (Data represent the mean ± SD). Statistical analysis was performed using one-way ANOVA. *p < 0.05, ***p < 0.001. **C** Lungs were isolated from WT and EMCN^ecko^ mice and lysed for Western blot analysis of the indicated proteins. Bands were quantified using ImageJ in **F**. Each experiment was repeated thrice, and the statistical significance of differences between gray intensities was determined using the unpaired Student’s t test. **D** S100A8/A9 staining in the lungs of WT and EMCN^ecko^ mice showed significant S100A8/A9-positive cell infiltration in the lungs of EMCN^ecko^ mice. **E** KEGG analyses of the significantly downregulated and upregulated genes in the lungs of WT and EMCN^ecko^ tumor-bearing mice (n = 3 biological replicates). **F** The mRNA levels of representative key factors were measured by quantitative PCR. RNA was extracted from the lungs of WT and EMCN^ecko^ mice inoculated with tumors (n = 3 biological replicates). **G** Neutrophils in the premetastatic lung (capable of reaching lungs but unable to grow up) and metastatic lung were determined by immunofluorescence staining in WT and EMCN^ecko^ mice. Scale bars, 75 μm (left). The number of Ly6G-positive cells was quantified by ImageJ (right)

Next, we attempted to clarify the steps of the metastatic cascade affected by EMCN defects. Based on our results indicating (i) no difference in primary tumor growth between WT and EMCN^ecko^ mice (Fig. 2B) and that (ii) EMCN deficiency increased lung metastasis (Fig. 2F and I) and pulmonary vascular permeability (Fig. 3D), we hypothesized that EMCN deletion might contribute to enhanced colonization of metastatic cells after changes in the host microenvironment, which relies on the formation of a lung metastatic niche. Vascular permeability is considered to be one of the important characteristics of the premetastatic niche [28] that plays an important role in tumor metastasis [29–31]. Transcriptome sequencing and protein detection were used to evaluate whether EMCN defects in endothelial cells could affect the formation of pulmonary premetastatic niches by affecting vascular permeability. MMP9, TGF-β and S100A8/A9 have been demonstrated to increase lung inflammation or immunosuppressive factors and play a key role in the initiation of the premetastatic niche [28, 32]. Next, transcriptome analysis of lung tissue was performed in EMCN^ecko^ mice, WT mice and tumor-bearing WT mice. We focused on the changes in premetastatic niche-related genes. Differentially expressed genes were identified as those upregulated or downregulated by > 1.5-fold in the groups of tumor-bearing WT mice vs. WT mice and EMCN^ecko^ vs. WT mice. We found that EMCN deficiency led to changes in the premetastatic niche in the lung, which was similar to the premetastatic niche induced by LLC tumors (Fig. 4A). Furthermore, EMCN deficiency was associated with changes affecting the host microenvironment, resulting in an increase in genes related to the premetastatic niche (TGFB, S100A8/A9, MMPs, etc.). The key factors in the premetastatic niche were detected, and the expression of S100A8/A9, MMP9 and TGF-β was increased in the lungs of EMCN^ecko^ mice. The expression levels of MMP9, TGF-β and S100A8/A9 were confirmed by Western blot and IHC, respectively (Fig. 4B–D). In brief, these results indicated that EMCN deficiency leads to the formation of a lung metastatic niche.

### EMCN deficiency promotes the recruitment of neutrophils to lung metastatic foci

It has been demonstrated that overexpression of EMCN can prevent neutrophil-endothelial cell interactions in vitro and inhibit the infiltration of CD45^+^ and NIMPR14^+^ (neutrophil marker) cells, but there were no changes in F4/80^+^ (monocyte/macrophage marker) cells [12]. An increasing number of studies have confirmed that neutrophils play an important role in the formation of a premetastatic niche in tumor lung metastasis [33, 34]. We analyzed the premetastatic niche lungs of WT and EMCN^ecko^ tumor-bearing mice by RNA-seq. The results showed that EMCN deletion affected the inflammatory response and neutrophil chemotaxis (Fig. 4E). The increase in cytokines was measured by qRT‒PCR (Fig. 4F). Therefore, we hypothesized that EMCN deficiency may lead to the aggregation of neutrophils in the lung, promote the formation of a premetastatic niche, and thereby promote growth and metastasis. We next detected neutrophil infiltration in the premetastatic niche and metastatic lung of WT and EMCN^ecko^ mice by immunofluorescence staining and found that Ly6G^+^ and S100A9^+^ (neutrophil marker) cells were significantly recruited in the lungs of EMCN^ecko^ tumor-bearing mice compared with WT tumor-bearing mice (Fig. 4G, Additional file 3: Fig. S3E). The above results led to the conclusion that EMCN deletion can cause neutrophil infiltration in vivo.

### EMCN deficiency promotes lung metastasis in a neutrophil-dependent manner

**Fig. 5:**
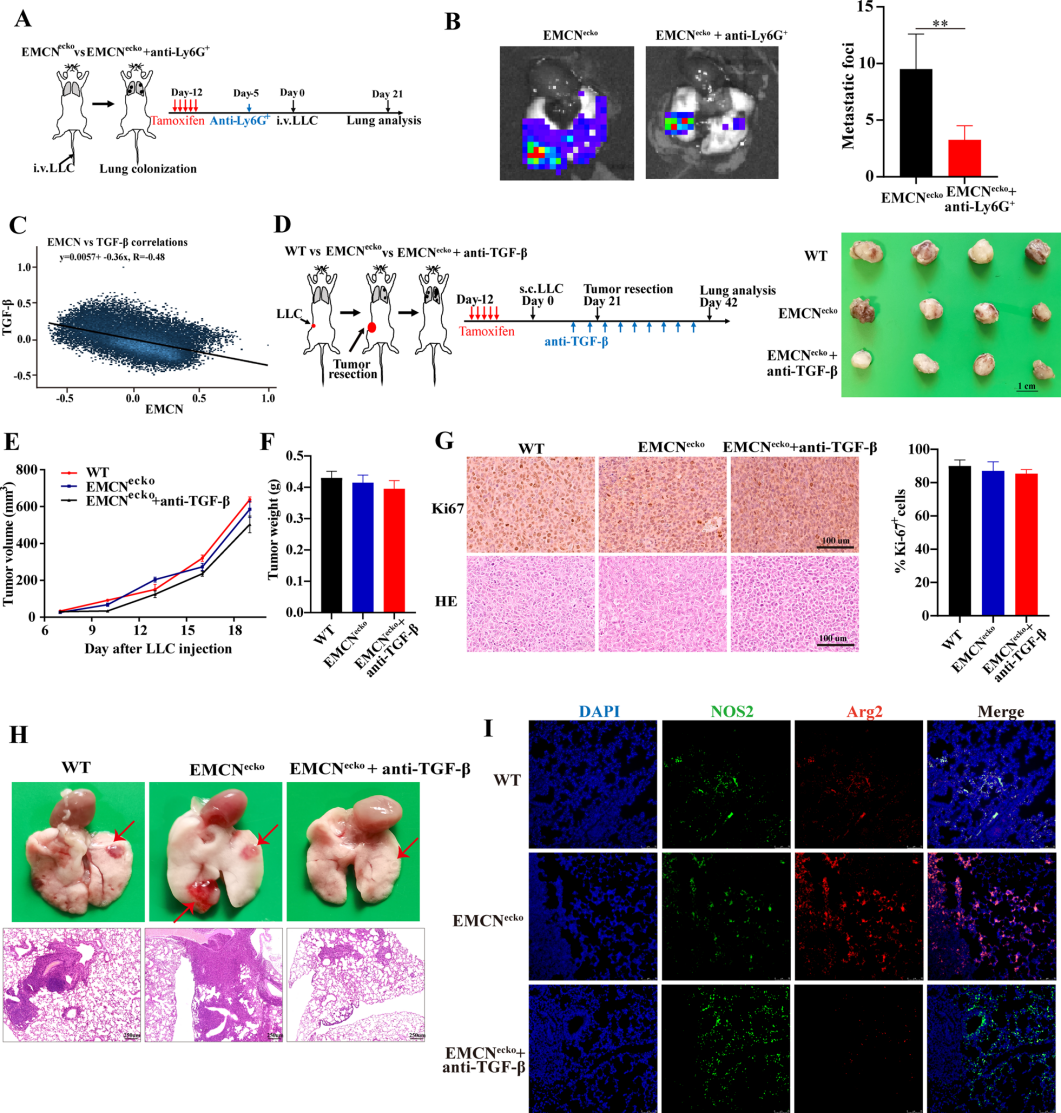
Anti-TGF-β inhibits tumor metastasis by inhibiting neutrophil polarization. **A** The schematic diagram shows the experimental design of tamoxifen induction, Anti-Ly6G^+^ antibody administration, intravenous injection of LLC tumor cells and lung imaging. **B** Representative lung images showing the diminished metastases to the lungs of EMCN^ecko^ mice (n = 4 per group) treated with anti-Ly6G^+^ antibody. Data represent the mean ± SD. Statistical analysis was performed using unpaired Student’s t test. **p < 0.01. **C** The association of TGF-β and EMCN in all normal specimens was analyzed using online datasets (https://hiplot.com.cn/advance/correlation-analyzer). **D** The schematic diagram shows the experimental design of tamoxifen induction, subcutaneous inoculation of LLC tumor cells, Anti-Ly6G^+^ injection, tumor resection and lung analysis (left). Photographs show the tumors on Day 21. **E** Tumor volumes and **F** tumor weights were measured for each group. **G** Representative Ki67 staining images of tumor sections are shown from WT and EMCN^ecko^ mice with and without anti-TGF-β injection (left panel). Quantification of Ki67 expression was performed (right panel) (n = 4 mice per group). Scale bars, 100 μm. **H** Representative images and HE staining of lung metastasis in WT or EMCN^ecko^ mice with or without anti-TGF-β injection. **I** Lung tissues were subjected to double-label immunofluorescence for NOS2 (green) and Arg2 (red). DAPI (blue): cell nuclei. N1 neutrophil marker (NOS2^+^) and N2 neutrophil marker (Arg2^+^). Scale bar: 75 μm

Next, we evaluated whether the increased neutrophils caused by EMCN deficiency are functional, revealing an important mechanism of lung metastasis. Neutrophils in vivo were depleted with Ly6G^+^ antibody before and after the tail vein injection of LLC cells into EMCN^ecko^ mice. Through the formation of lung metastasis foci and in vivo imaging, we found that neutrophil depletion reduced lung metastasis in vivo (Fig. 5A and B). In brief, these results demonstrated that Ly6G^+^ neutrophils play an important role in lung metastasis caused by EMCN deficiency.

### Anti-TGF-β suppresses lung metastasis by blocking TGF-β-induced N2 neutrophil polarization

A deeper understanding of the metastasis process is needed to develop new therapies for cancer patients. We assessed whether the increased TGF-β was functionally important in LLC lung metastasis of EMCN^ecko^ mice. It has been reported that the polarization of N2 neutrophils promotes tumor metastasis of breast and gastric cancers [35, 36]. TGF-β has been shown to play an important role in inducing N1 to N2 polarization [37]. Through bioinformatics analysis of TCGA dataset and experimental verification, we found a negative correlation between EMCN and TGF-β (Figs. 4C and 5C). Therefore, we hypothesized that EMCN deletion led to an increase in TGF-β in the microenvironment, promoted the polarization of N1 neutrophils into N2 neutrophils, and thereby promoted lung metastasis. Therefore, specific blockade of TGF-β-induced neutrophil polarization is a meaningful therapeutic strategy to treat lung metastasis.

To further assess the anti-TGF-β suppressive effect of neutrophil polarization, we injected LLC cells subcutaneously into WT and EMCN^ecko^ mice. EMCN^ecko^ mice were treated with or without anti-TGF-β antibody and examined for N1-/N2-associated markers. Consistent with the above-described results (Fig. 2B), EMCN deletion did not affect growth but promoted lung metastasis of primary tumors in the WT and EMCN^ecko^ groups (Fig. 5G and H). However, compared with EMCN^ecko^ mice, anti-TGF-β antibody did not affect tumor growth but inhibited lung metastasis caused by EMCN deletion (Fig. 5E–H and Additional file 3: Fig S3F). We also examined the expression of relevant N1- and N2-neutrophil markers in lung metastatic tumors by immunofluorescence analysis and found that EMCN^ecko^ mice could induce N2 neutrophils (Arg2^+^) to infiltrate metastatic tumors. However, anti-TGF-β antibody significantly inhibited N2 neutrophils, and N1 neutrophil infiltration was primarily observed (Fig. 5I and Additional file 3: Fig S3G). Taken together, these results strongly suggest that the anti-TGF-β antibody is a promising therapeutic target that can be used to prevent or treat lung metastasis caused by neutrophil polarization.

### Notch inhibitor suppresses tumor growth and metastasis by upregulating EMCN

**Fig. 6:**
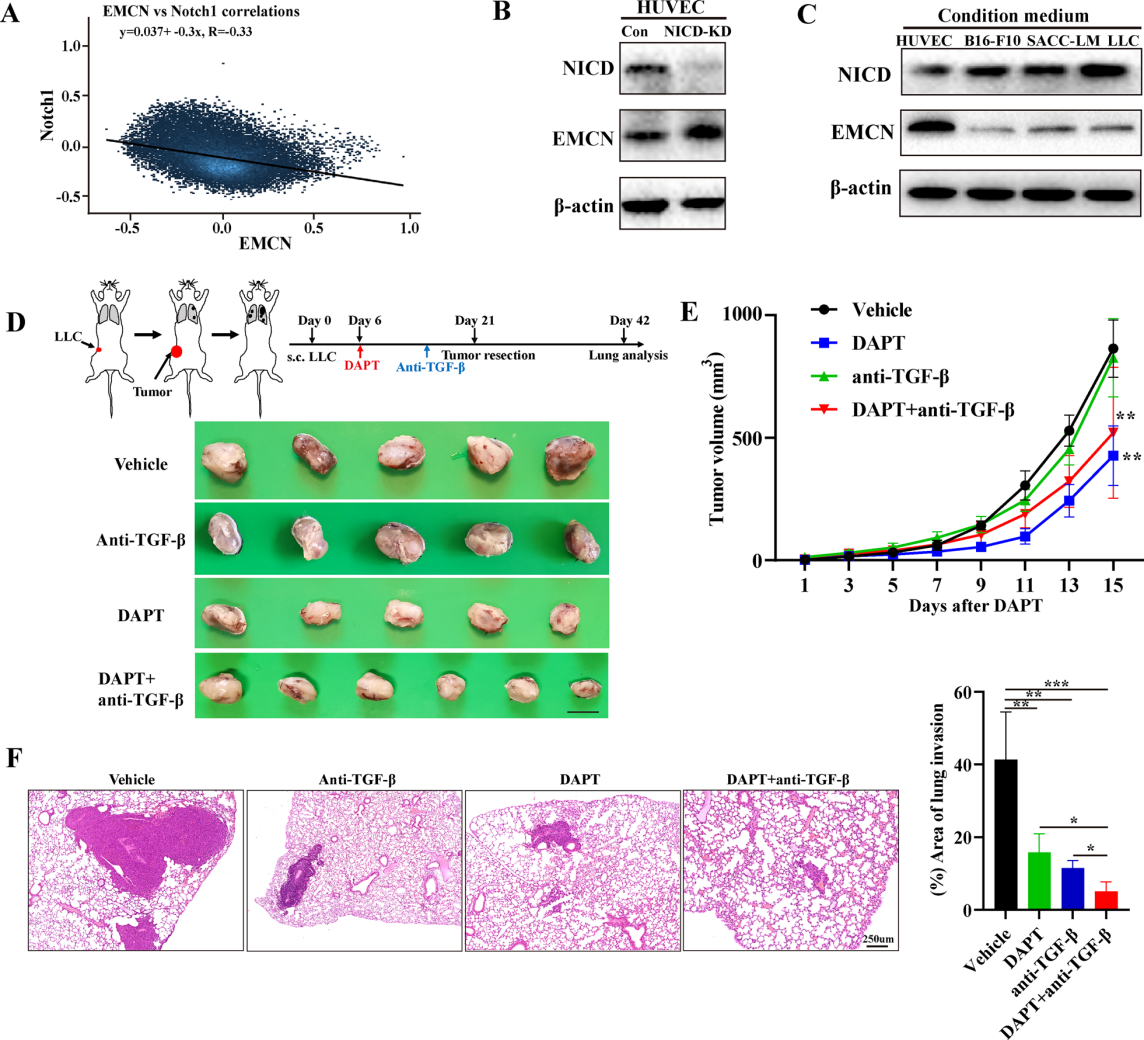
Notch inhibition upregulated EMCN expression in HUVECs, and combination therapy with DAPT and anti-TGF-β inhibited lung metastasis. **A** The association of TGF-β and EMCN in all specimens was analyzed using online datasets (https://hiplot.com.cn/advance/correlation-analyzer). **B** The efficiency of NICD knockdown in HUVECs was measured by Western blotting, and NICD downregulation increased EMCN protein levels in HUVECs. **C** HUVECs were cocultured with B16-F10, ACC-LM and LLC cell conditioned medium for 48 h. NICD and EMCN expression levels in HUVECs were detected by Western blotting. **D** Schematic diagram showing the experimental procedure of subcutaneous implantation of LLC tumor cells, DAPT or anti-TGF-β injection, tumor resection and lung analysis (upper). Representative images of tumors harvested from WT mice with vehicle or DAPT after 21 days of LLC tumor implantation (below). **E** Growth curves of LLC tumors in WT mice treated with vehicle, DAPT alone, anti-TGF-β antibody alone, or a combination of anti-TGF-β antibody with DAPT. Tumors were measured by a caliper every other day. n = 5 mice per group. **p < 0.01, two-way ANOVA. **F** H&E sections of mouse lungs 3 weeks after subcutaneous tumor resection treated with vehicle, DAPT alone, anti-TGF-β antibody alone, or the combination of anti-TGF-β antibody with DAPT (left). Histological quantification of the area of lung invasion (right). *p < 0.05, **p < 0.01, ***p < 0.001

Previous studies have identified the key regulatory relationship of the Notch signaling pathway in the regulation of EMCN expression in hepatic ischemia/reperfusion injury. Pharmaceutical Notch blockade dramatically upregulated EMCN and prevented trans-endothelial migration of neutrophils in vitro. Furthermore, in RBPj (integrated transcription factor of typical Notch signaling) knockout mice, the Notch signal was inactivated, but the expression of EMCN was upregulated [15]. Through bioinformatics analysis of TCGA data, an obvious negative correlation was noted between EMCN and Notch1 (Fig. 6A). To further investigate whether Notch regulates EMCN expression, EMCN protein expression was detected by inhibiting NICD (a key protein of the Notch signaling pathway). The results showed that NICD inhibition significantly increased the expression level of EMCN in HUVECs (Fig. 6B and Additional file 4: Fig. S4A). After we cocultured HUVECs with different tumor cell-conditioned media, the results showed that tumor cells significantly activated NICD in HUVECs, which was consistent with previous studies [9]. Interestingly, NICD activation significantly inhibited the expression of EMCN on HUVECs (Fig. 6C and Additional file 4: Fig. S4B). Accordingly, we used the Notch pathway inhibitor DAPT, a γ-secretase inhibitor, to detect its effect on tumor growth and metastasis using a syngeneic mouse model. To determine whether DAPT can inhibit tumor growth and metastasis, we treated tumor-bearing mice with DAPT or vehicle. The results showed that DAPT slightly inhibited tumor growth and metastasis compared with the vehicle group (Fig. 6D–F). Immunohistochemistry results for Ki67 staining confirmed these findings (Additional file 4: Fig. S4C and 4D). Western blot analysis showed that EMCN expression was significantly upregulated in the lungs of DAPT-treated mice compared with that in the vehicle-treated mice (Additional file 4: Fig. S4E). These results confirmed that DAPT could inhibit tumor growth and elicit a suppressive effect on lung metastases by upregulating EMCN.

### Combined treatment with a Notch inhibitor and an anti-TGF-β antibody synergistically reduces tumor growth and metastasis

TGF-β weakens the intrinsic antitumor potential of immune cells in the tumor microenvironment by increasing the inhibitory effect on key immune cells of innate and adaptive immunity. Therefore, the antitumor response of myeloid cells and lymphocytes is hypothesized to be enhanced by inhibiting TGF-β [38]. It is worth mentioning that due to the limited clinical efficacy of TGF-β inhibitor monotherapy, a large number of combined therapies are being assessed, such as combined cytotoxic drugs, radiotherapy, and immune checkpoint inhibitors [39–42]. In light of the data described above, DAPT has a good therapeutic effect on tumor growth and metastasis in syngeneic mice, whereas the use of DAPT as a Notch signaling pathway inhibitor in combination with anti-TGF-β antibody has not been reported. We again used the mouse tumor metastasis model of syngeneic subcutaneous tumor grafting and surgical resection. Once tumors are palpable, the animals were assigned to different treatment systems as shown in Fig. 6D. Combined DAPT and anti-TGF-β administration did not significantly affect mouse body weight or damage to important organs compared with control mice, indicating that the regimen with a low dose of DAPT and anti-TGF-β is relatively safe (Additional file 5: Fig. S5A and B). The growth of subcutaneous tumors was significantly inhibited (Fig. 6D and E). Immunohistochemical analysis of Ki67 revealed differences in proliferation (Additional file 4: Fig. S4C). Tumor metastasis was found in the lung metastasis foci and HE-stained sections (Fig. 6F, Additional file 4: Fig. S4D). Notably, we found that the combination of DAPT and anti-TGF-β antibody dramatically reduced the area of the metastatic nodules and slightly inhibited tumor growth compared to the DAPT group (Fig. 6F). We used a second animal model to confirm the same results (Additional file 5: Fig. S5C and D). The survival rate of mice was significantly improved (Additional file 5: Fig. S5E). Taken together, we demonstrated the important role of DAPT combined with anti-TGF-β antibody in tumor growth and metastasis, especially the significant inhibition of tumor metastasis and improved survival using a syngeneic mouse model.

### The clinical significance of EMCN and Notch expression in cancer patients from TCGA

To further validate our experimental results, we examined the clinical significance of Notch1 and EMCN in lung cancer by analyzing TCGA dataset. We evaluated the relationship between Notch1 and EMCN expression levels and patient survival. The results showed that patients with high EMCN expression exhibited a higher survival rate, whereas patients with high Notch1 expression exhibited a significantly lower survival rate (Additional file 5: Fig. S5F).

**Fig. 7:**
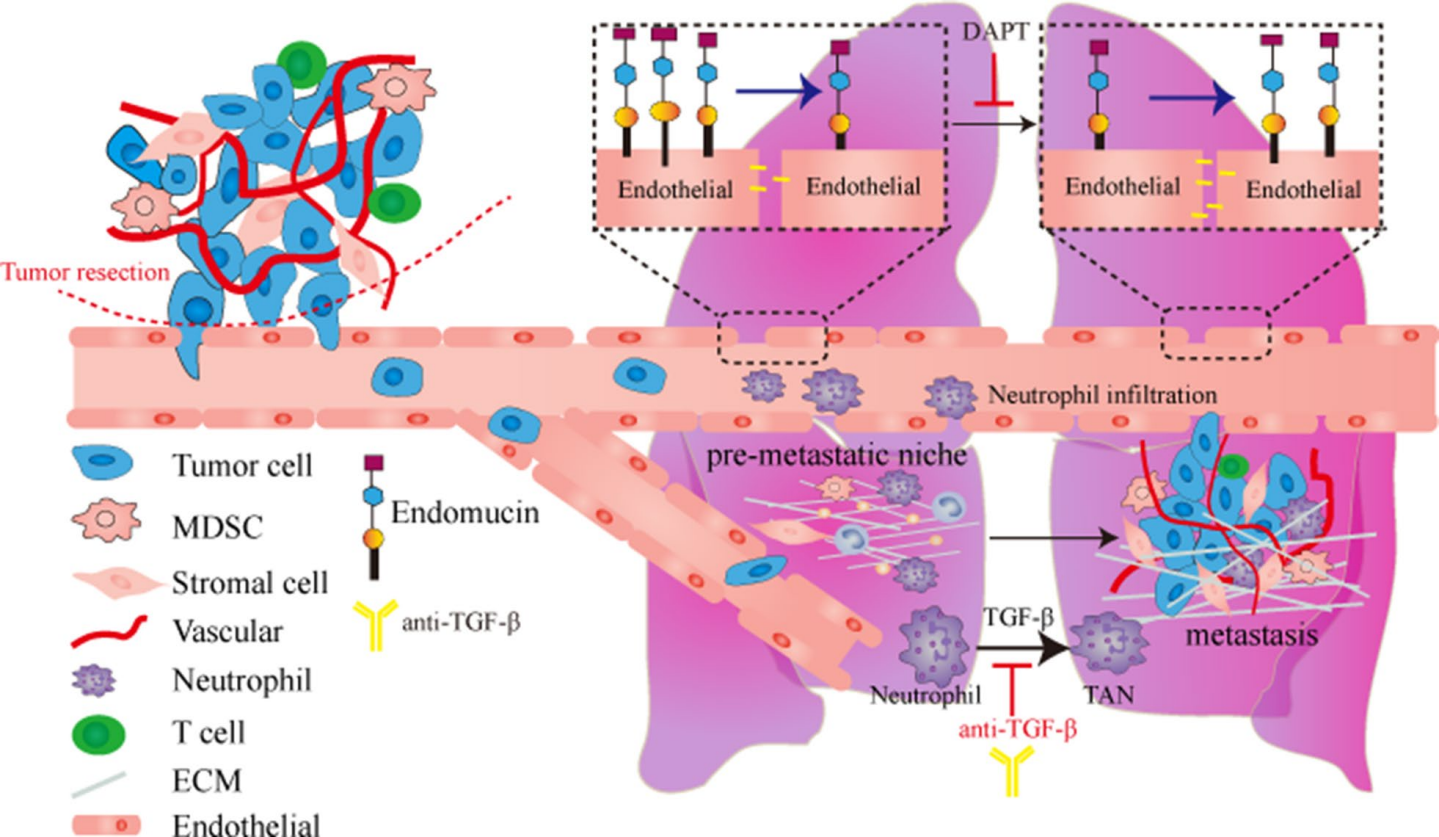
A proposed working model. The loss of endothelial EMCN caused an increase in vascular permeability, produced a premetastatic niche and promoted tumor lung metastasis. Downregulation of EMCN expression was reversed by treatment with a Notch inhibitor combined with an anti-TGF-β antibody to inhibit lung metastasis

In conclusion, we demonstrate a new antimetastatic effect of EMCN on lung metastasis. EMCN deletion caused a remarkable increase in metastasis by affecting the formation of the premetastatic niche. TGF-β in the premetastatic niche promoted the polarization of neutrophils and exacerbated lung metastasis. Pharmacological inhibition of Notch improved EMCN expression, inhibited tumor metastasis, and showed additive effects when combined with anti-TGF-β antibody therapy. Taken together, we demonstrate a possible new therapeutic strategy involving Notch inhibitors and anti-TGF-β antibodies in clinical tumor patients (Fig. 7).

## Discussion

Our data from LLC primary tumors between WT and EMCN^ecko^ mice revealed no differences in primary tumor growth and blood vessel formation. This finding led us to study the metastasis process downstream of the primary tumor and alterations in vascular function. We found higher EMCN expression levels in the lung compared with other tissues, including the heart, liver and spleen. We explored the hypothesis that EMCN knockout mice had increased lung colonization following tail vein injection of LLC cells compared to wild-type mice in part due to increased extravasation and increased permeability of pulmonary vessels, which subsequently leads to alterations in the premetastatic niche. These results provide insight into how EMCN expression in endothelial cells affects tumor metastasis.

However, identifying specific EMCN downstream molecular changes that are critical for metastasis can be very difficult. This challenge becomes further exacerbated when we consider that EMCN is not expressed on tumor cells but on normal endocytic endothelial cells. It has been found that the combination of the tumor cell surface ligand app and endothelial cell surface receptor DR6 can lead to programmed death of endothelial cells and promote tumor metastasis [22]. Therefore, EMCN on endothelial cells may interact with ligands on various stroma and tumor cells. However, previous studies have found that EMCN, as a member of the mucin family, is a sialylated glycoprotein and has more anti-adhesion properties [43]. To date, no ligands that bind to EMCN have been identified. Many studies have demonstrated the role of EMCN in endothelial cell angiogenesis [44]. Although we did not observe significant differences in angiogenesis in vivo, this does not exclude the role of EMCN in the vascular function of endothelial cell formation. Few studies have found that molecules on endothelial cells can affect endothelial permeability and tumor metastasis [45]. Therefore, we demonstrated the important role of EMCN deletion in endothelial cell infiltration and tumor premetastatic niche formation.

Lung metastasis is commonly observed in different types of cancer, including breast cancer, gastrointestinal tumors, melanoma and different types of sarcomas in addition to lung cancer itself [46]. In lung metastasis of tumors, TGF-β can induce the secretion of ANGPTL4. These secreted mediators enhance the extravasation of tumor cells in the lung by weakening the cell‒cell connection between endothelial cells [47]. We found changes in TGF-β levels in the lungs of WT and EMCN^ecko^ tumor-bearing mice. TGF-β is critical for immunosuppression in the tumor microenvironment. It inhibits the function of many components of the immune system and promotes tumor occurrence. Recent studies have shown that TGF-β plays a role in tumor immune escape and adverse responses to tumor immunotherapy [48, 49]. The unique function and regulation of neutrophils in cancer are closely related to the formation of lung premetastatic niches in tumor-bearing mice [50]. An interesting difference we observed was the increase in neutrophils in EMCN^ecko^ premetastatic lung and tumor-bearing lung. Because EMCN has been shown to prevent leukocytes from adhering to endothelial cells [12], EMCN deletion may affect neutrophil recruitment to the lung tissue. Neutrophils in the lung play a role in forming a premetastatic niche and promoting tumor metastasis after polarization [36]. We demonstrated that lung neutrophils lacking EMCN were largely increased and induced to form N2 neutrophils by TGF-β in the microenvironment to promote tumor metastasis and growth. After neutralizing TGF-β by injecting a TGF-β antibody in vivo, the inhibitory effect of the N2 neutrophil phenotype was observed.

Although our research focuses on primary tumors and lung metastasis, there are additional problems that need to be discussed. Compared with WT, the lung lacking EMCN may or may not be the only organ that provides a favorable premetastatic niche for tumor cell colonization. For example, whether EMCN defects in the liver provide a favorable premetastatic niche for the liver metastasis of mouse colorectal cancer cells warrants further study using a liver metastasis model of colorectal cancer. We confirmed that EMCN affects vascular function, but we did not further study the other biological functions of EMCN on endothelial cells, such as endothelial cell senescence and programmed death.

Preclinical studies have shown that TGF-β inhibition combined with checkpoint inhibitors can significantly enhance its immune effect, whereas TGF-β inhibition may show limited efficacy as a monotherapy [51, 52]. In another combined application study, radiotherapy combined with blocking TGF-β antibody enhanced the systemic antitumor response [53]. A series of Notch signaling pathway inhibitors have been tested in phase I/II clinical trials of various types of cancer, and the complexity of notch inhibition and alternative carcinogenic signals that may be provided by other pathways have produced more adverse reactions [54]. Based on our experimental data, the growth and metastasis of mouse syngeneic tumors was inhibited by TGF-β antibody combined with DAPT. However, bioinformatics analysis of limited clinical data showed that although the difference was not highly significant, the survival rate of patients with high expression of EMCN and low expression of Notch was significantly increased. Our combined therapy research in mice provides a new strategy for the clinical prevention and treatment of tumor metastasis.

In conclusion, we demonstrate that EMCN deficiency in endothelial cells promotes metastasis by providing a suitable premetastatic niche for cancer cell extravasation and lung colonization. Therefore, targeting notch-mediated upregulation of EMCN in endothelial cells combined with TGF-β inhibition may represent a new method to prevent or treat metastasis. Future extensive studies should determine the value of EMCN expression as a potential prognostic biomarker in patients with melanoma and other highly metastatic cancers.

## Supplementary Information

**Additional file 1: Figure S1.** The downregulation of EMCN expression is related to metastasis and recurrence in breast cancer patients. **(A)** Heatmap of differentially expressed genes in breast cancer from TCGA datasets. **(B)** Volcano plot of differentially expressed mRNAs. Red dots represent upregulated mRNAs, and blue dots represent downregulated mRNAs. (C) EMCN expression in adjacent tissues and tumor tissues was analyzed (Wilcox tests, ****p < 0.0001, **p < 0.01, **p < 0.05). (D) EMCN gene expression was assessed in metastatic patients and normal tissues from TCGA dataset (Wilcox tests, ****p < 0.0001). (E) EMCN gene expression levels in recurrence patients and normal tissues from TCGA dataset (Wilcox tests,****p < 0.0001).

**Additional file 2: Figure S2.** Identification of genotype and knockout efficiency. **(A)** EMCN^loxp/loxp^ and Tek-creERT2 were identified by PCR. **(B)** Lung, liver, kidney and spleen extracts of WT and EMCNecko mice were probed for EMCN protein expression. **(C)** HE sections of lung, liver, kidney and spleen in WT and EMCN^ecko^ mice. **(D)** Lung metastasis area of mice at different times. **(E)** Lung metastasis was detected 15 days after subcutaneous tumor resection of melanoma.

**Additional file 3: Figure S3.** Knockdown efficiency of EMCN in HUVECs and the effect of EMCN on proliferation and metastasis. **(A)** qRT-PCR for the detection of EMCN mRNA in HUVECs infected with shRNA lentivirus that targets EMCN compared with HUVECs infected with a control shRNA. **(B)** Proliferation assays at 24, 48, 72 h and 96 h after plating. HUVECs with reduced EMCN expression display significantly less proliferative potential than control cells. **(C)** EMCN protein expression in naive lung, premetastatic lung and metastatic lung (left). **(D)** Representative image of lung sections showing extravasation rhodamine B-dextran (red) after intravenous injection of rhodamine B-dextran. **(E)** Neutrophils (S100A9) in the premetastatic lung (capable of reaching lungs but unable to grow up) were assessed by immunofluorescence staining in WT and EMCN^ecko^ mice. **(F)** Statistical analysis of lung metastasis in different groups. (G) Quantification of immunofluorescence staining for N1 (NOS2^+^) and N2 (Arg2^+^) neutrophils for the panel.

**Additional file 4: Figure S4.** The combination of anti-TGF-β antibody with DAPT synergistically suppresses tumor growth and metastasis. **(A) and (B)** Grayscale values of NICD and EMCN were quantified by ImageJ. **(C)** Representative Ki67 staining images of tumor sections from each group are shown from WT mice by IHC. **(D)** Representative photographs were obtained from the lungs of LLC-injected WT mice treated with vehicle, DAPT alone, anti-TGF-β antibody alone, or a combination of anti-TGF-β antibody with DAPT. **(E)** EMCN protein levels in the lung were significantly increased after DAPT injection.

**Additional file 5: Figure S5.** Toxicity of combined therapy and survival time of mice.** (A)** HE staining of liver, kidney and intestine from the vehicle and the combination of DAPT and anti-TGF-β antibody groups. **(B)** Body weight changes of LLC tumor-bearing mice treated with vehicle or the combination of DAPT and anti-TGF-β antibody. **(C)** The weight of subcutaneous tumors (B16-F10) in mice was measured (Left, *p < 0.05). **(D)** The number of B16-F10 lung metastasis foci in mice was analyzed (right, ***p < 0.001). **(E)** Mice were randomly divided into two groups (n = 6) and treated with solvent or DAPT and anti-TGF-β antibody daily until subcutaneous tumor resection. The left side indicates LLC cell inoculation, and the right side indicates B16-F10 cell inoculation. Kaplan‒Meier survival analysis showed prolonged survival for the DAPT-treated lung metastasis model mice. Log-rank **p < 0.01 compared with solvent-treated controls. **(F)** The EMCN and Notch1 expression level was significantly correlated with patient survival.

**Additional file 6: Table 1.** Sequences for primers for qRT-PCR and target sequences of shRNA.

**Additional file 7.: Table 2.** The sequencing coverage and quality statistics for each sample are summarized.

## Data Availability

Only publicly available data were used in this study, and the data sources and handling of these data are described in the Materials and Methods. Further information is available from the corresponding author upon request.

## Abbreviations

CCK-8: Cell counting kit-8
DAPT: γ-Secretase inhibitor
DEGs: Differentially expressed genes
EMCN: Endomucin
EMCN^ecko^: Endothelial cell knockout EMCN
HUVEC: Human umbilical vein endothelial cells
IF: Immunofluorescence
IHC: Immunohistochemistry
MMP-9: Matrix metalloproteinase 9
NICD: Notch intracellular fragment
qRT‒PCR: Quantitative reverse transcription-polymerase chain reaction
S100A8/A9: S100 calcium-binding protein A8/A9
TCGA: The Cancer Genome Atlas
TGF-β: Transforming growth factor-β
WT: Wide type
ZO-1: Zonula occludens 1
