# Supplementary figures and images for: Loss of endothelial EMCN drives tumor lung metastasis through the premetastatic niche
Source: J Transl Med. 2022 Oct 2;20:446. doi: 10.1186/s12967-022-03649-4 (PMC9528146; doi:10.1186/s12967-022-03649-4)

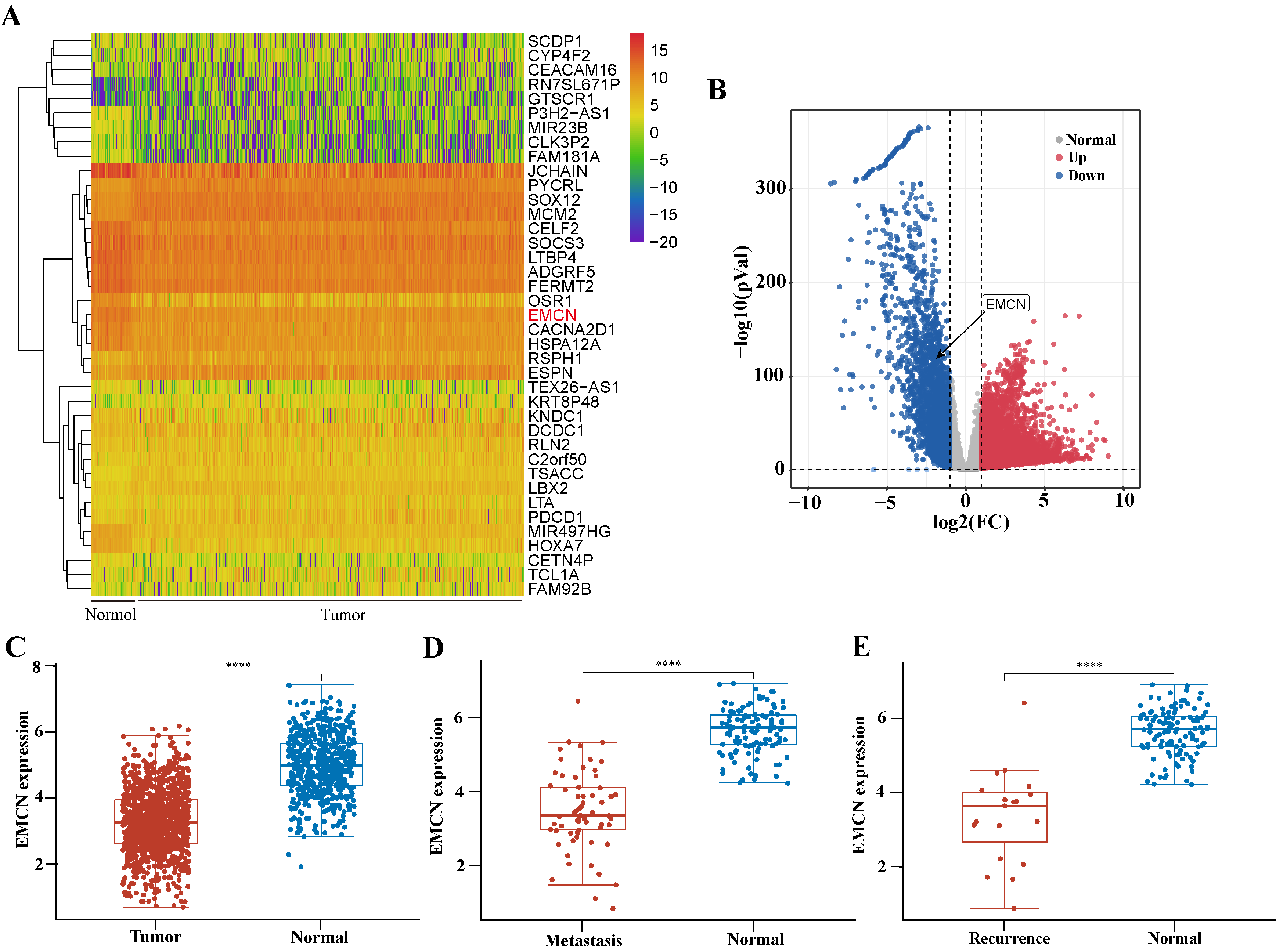

Supplement: Supplementary file 1 — Additional file 1: Figure S1. The downregulation of EMCN expression is related to metastasis and recurrence in breast cancer patients. (A) Heatmap of differentially expressed genes in breast cancer from TCGA datasets. (B) Volcano plot of differentially expressed mRNAs. Red dots represent upregulated mRNAs, and blue dots represent downregulated mRNAs. (C) EMCN expression in adjacent tissues and tumor tissues was analyzed (Wilcox tests, ****p < 0.0001, **p < 0.01, **p < 0.05). (D) EMCN gene expression was assessed in metastatic patients and normal tissues from TCGA dataset (Wilcox tests, ****p < 0.0001). (E) EMCN gene expression levels in recurrence patients and normal tissues from TCGA dataset (Wilcox tests,****p < 0.0001). [file 12967_2022_3649_MOESM1_ESM.tif]

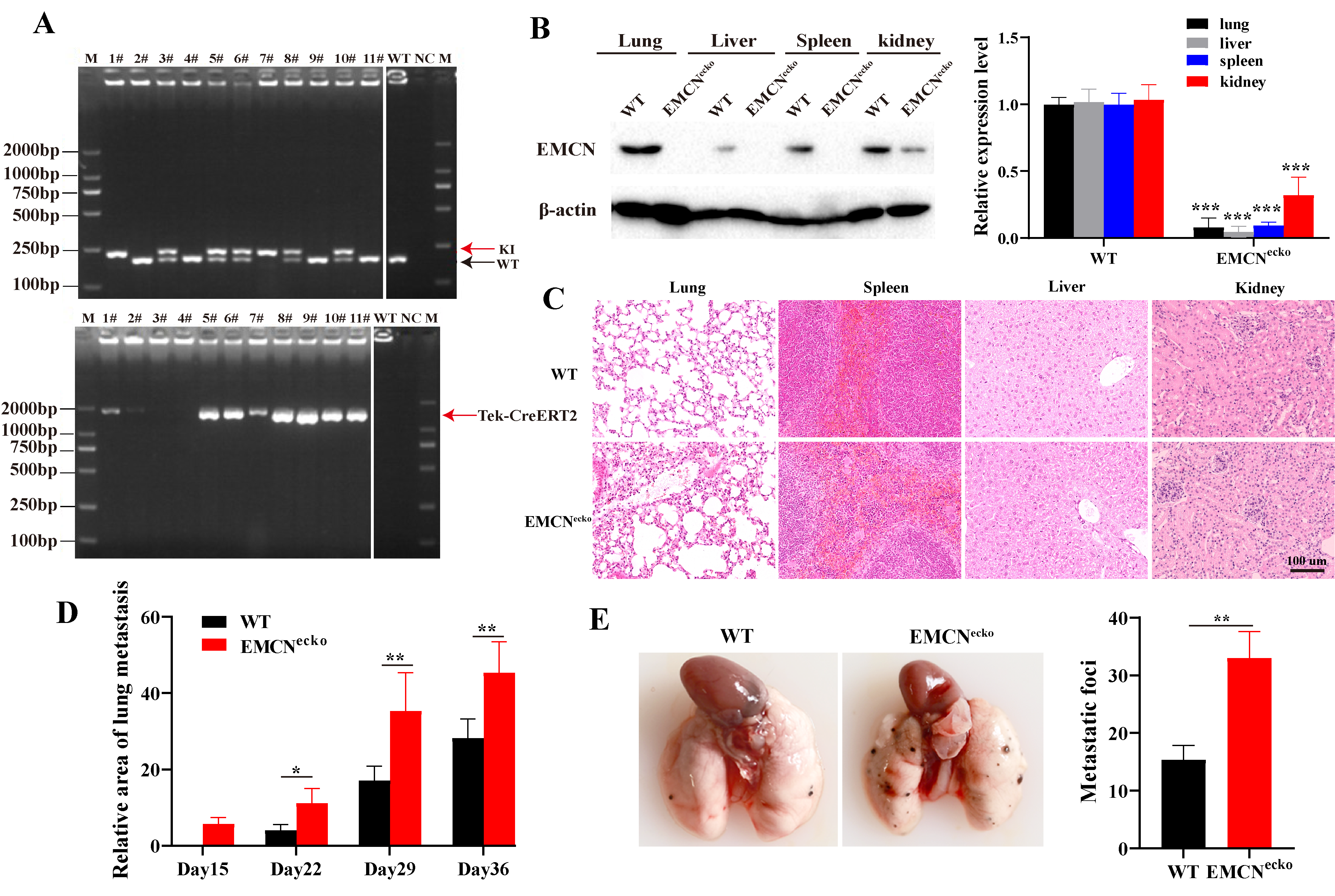

Supplement: Supplementary file 2 — Additional file 2: Figure S2. Identification of genotype and knockout efficiency. (A) EMCNloxp/loxp and Tek-creERT2 were identified by PCR. (B) Lung, liver, kidney and spleen extracts of WT and EMCNecko mice were probed for EMCN protein expression. (C) HE sections of lung, liver, kidney and spleen in WT and EMCNecko mice. (D) Lung metastasis area of mice at different times. (E) Lung metastasis was detected 15 days after subcutaneous tumor resection of melanoma. [file 12967_2022_3649_MOESM2_ESM.tif]

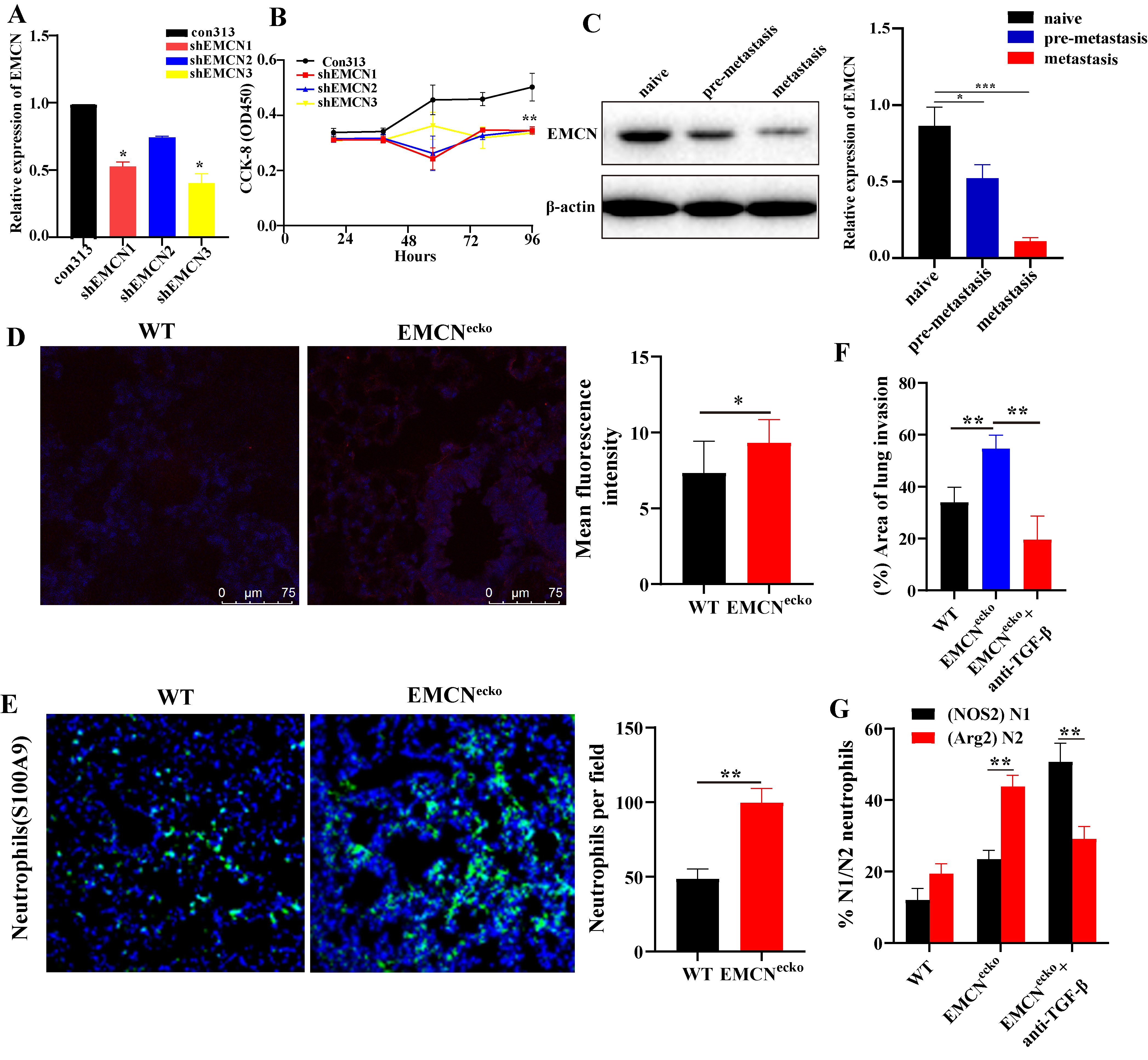

Supplement: Supplementary file 3 — Additional file 3: Figure S3. Knockdown efficiency of EMCN in HUVECs and the effect of EMCN on proliferation and metastasis. (A) qRT-PCR for the detection of EMCN mRNA in HUVECs infected with shRNA lentivirus that targets EMCN compared with HUVECs infected with a control shRNA. (B) Proliferation assays at 24, 48, 72 h and 96 h after plating. HUVECs with reduced EMCN expression display significantly less proliferative potential than control cells. (C) EMCN protein expression in naive lung, premetastatic lung and metastatic lung (left). (D) Representative image of lung sections showing extravasation rhodamine B-dextran (red) after intravenous injection of rhodamine B-dextran. (E) Neutrophils (S100A9) in the premetastatic lung (capable of reaching lungs but unable to grow up) were assessed by immunofluorescence staining in WT and EMCNecko mice. (F) Statistical analysis of lung metastasis in different groups. (G) Quantification of immunofluorescence staining for N1 (NOS2+) and N2 (Arg2+) neutrophils for the panel. [file 12967_2022_3649_MOESM3_ESM.tif]

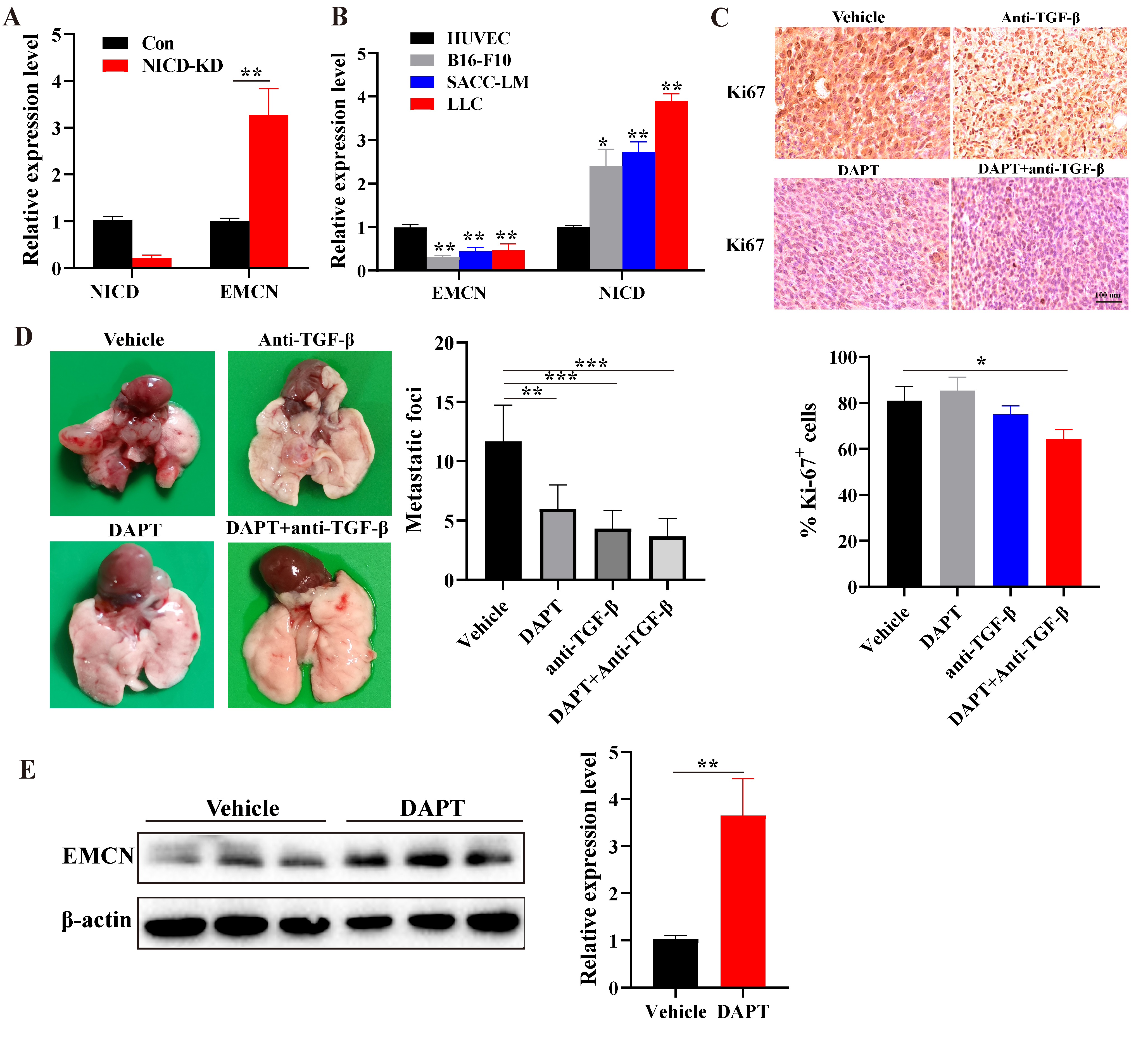

Supplement: Supplementary file 4 — Additional file 4: Figure S4. The combination of anti-TGF-β antibody with DAPT synergistically suppresses tumor growth and metastasis. (A) and (B) Grayscale values of NICD and EMCN were quantified by ImageJ. (C) Representative Ki67 staining images of tumor sections from each group are shown from WT mice by IHC. (D) Representative photographs were obtained from the lungs of LLC-injected WT mice treated with vehicle, DAPT alone, anti-TGF-β antibody alone, or a combination of anti-TGF-β antibody with DAPT. (E) EMCN protein levels in the lung were significantly increased after DAPT injection. [file 12967_2022_3649_MOESM4_ESM.tif]

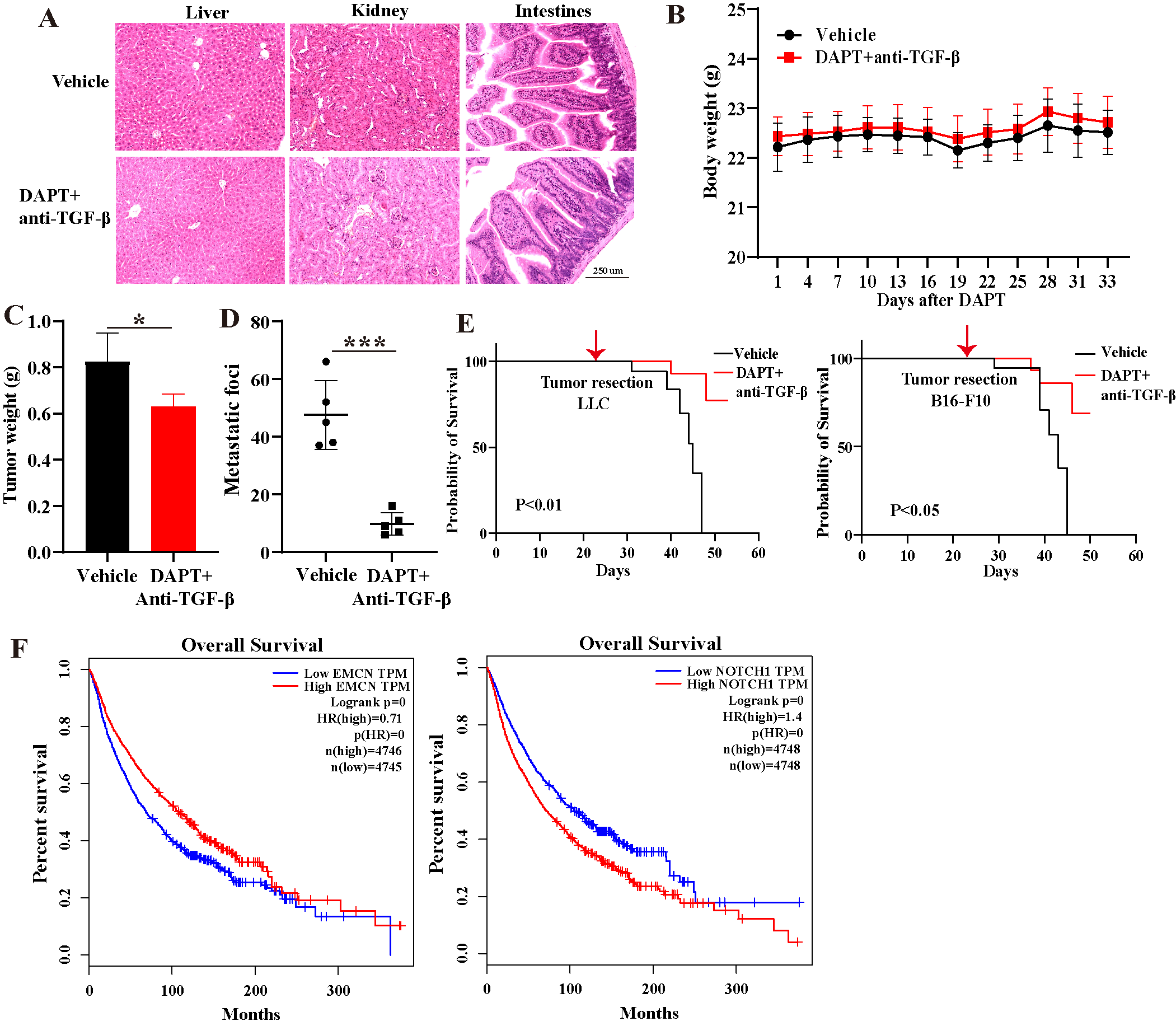

Supplement: Supplementary file 5 — Additional file 5: Figure S5. Toxicity of combined therapy and survival time of mice. (A) HE staining of liver, kidney and intestine from the vehicle and the combination of DAPT and anti-TGF-β antibody groups. (B) Body weight changes of LLC tumor-bearing mice treated with vehicle or the combination of DAPT and anti-TGF-β antibody. (C) The weight of subcutaneous tumors (B16-F10) in mice was measured (Left, *p < 0.05). (D) The number of B16-F10 lung metastasis foci in mice was analyzed (right, ***p < 0.001). (E) Mice were randomly divided into two groups (n = 6) and treated with solvent or DAPT and anti-TGF-β antibody daily until subcutaneous tumor resection. The left side indicates LLC cell inoculation, and the right side indicates B16-F10 cell inoculation. Kaplan‒Meier survival analysis showed prolonged survival for the DAPT-treated lung metastasis model mice. Log-rank **p < 0.01 compared with solvent-treated controls. (F) The EMCN and Notch1 expression level was significantly correlated with patient survival. [file 12967_2022_3649_MOESM5_ESM.tif]
