# Supplementary material for: Loss of endothelial EMCN drives tumor lung metastasis through the premetastatic niche
Source: J Transl Med. 2022 Oct 2;20:446. doi: 10.1186/s12967-022-03649-4 (PMC9528146; doi:10.1186/s12967-022-03649-4)
Supplement: Supplementary file 6 — Additional file 6: Table 1. Sequences for primers for qRT-PCR and target sequences of shRNA. [file 12967_2022_3649_MOESM6_ESM.pdf]

Gene	Target sequence
Negative Control(shNC)	TTCTCCGAACGTGTCACGT
ShEMCN1#(93325-1)	GCGTGAAGCTTCTTACCGTTA
ShEMCN2#(93326-1)	GACTCCATCATTTCAAACGTA
ShEMCN3#(93327-1)	CAACACCAAACACAGAATCAT

Primers for quantitative Real-Time PCR

gene	Forward	Reverse
hEMCN	5’-TGCAGGACTTTCTCCTTTTC-3’	5’-ATTTGTTCTGGTGGGTTTGT-3’
mEMCN	5’-AATACCAGGCATCGTGTCAGT-3’	5’-CTGATTCTCAGTCTTGTTCTGGG-3’
hGAPDH	5’-CAAATTCCATGGCACCGTCA-3’	5’-GGAGTGGGTGTCGCTGTTGA-3’
mGAPDH	5’-GTGGCAAAGTGGAGATTGCC-3’	5’-GATGATGACCCGTTTGGCTCC-3’
S100A8	5’-TGAGTGTCCTCAGTTTGTGCAG-3’	5’-TGTGAGATGCCACACCCACTTT-3’
S100A9	5’-CCTCACCATATGGCTCGGAC -3’	5’-ACGACTGCAAGATTGGAGCA-3’
MMP9	5’-GGACCCGAAGCGGACATTG-3’	5’-CGTCGTCGAAATGGGCATCT-3’
TGF-β	5’-GAGCCCGAAGCGGACTACTA-3’	5’-TGGTTTTCTCATAGATGGCGTTG-3’
CXCL1	5’-ACCGAAGTCATAGCCACACT-3’	5’-GTGCCATCAGAGCAGTCTGT-3’
CXCL3	5’-CAGCCACACTCCAGCCTA-3’	5’-CACAACAGCCCCTGTAGC-3’
TNF-α	5’-CAGGCGGTGCCTATGTCTC-3’	5’-CGATCACCCCGAAGTTCAGTAG-3’
