# Supplementary material for: Loss of endothelial EMCN drives tumor lung metastasis through the premetastatic niche
Source: J Transl Med. 2022 Oct 2;20:446. doi: 10.1186/s12967-022-03649-4 (PMC9528146; doi:10.1186/s12967-022-03649-4)
Supplement: Supplementary file 7 — Additional file 7.: Table 2. The sequencing coverage and quality statistics for each sample are summarized. [file 12967_2022_3649_MOESM7_ESM.pdf]

Quality statistic of different samples

**Sample**	**RawReads**	**RawBases**	**ValidBases**	**Q30**	**GC**
EMCN-L1	48.88M	7.33G	91.23%	90.57%	49.75%
EMCN-L2	49.37M	7.41G	91.53%	90.37%	49.75%
EMCN-L3	50.97M	7.65G	91.86%	90.50%	49.44%
EMCN-LLC-L1	49.78M	7.47G	91.63%	90.00%	49.32%
EMCN-LLC-L2	51.27M	7.69G	91.36%	89.66%	49.54%
EMCN-LLC-L3	47.28M	7.09G	90.90%	89.82%	49.51%
WT-L1	48.30M	7.25G	91.38%	90.29%	49.03%
WT-L2	50.10M	7.51G	91.46%	90.54%	49.20%
WT-L3	51.02M	7.65G	91.64%	90.28%	48.75%
WT-LLC-L1	47.67M	7.15G	91.85%	90.15%	48.77%
WT-LLC-L2	48.66M	7.30G	91.68%	90.17%	49.39%
WT-LLC-L3	48.06M	7.21G	91.19%	89.51%	49.06%
Con313	50.01M	7.52G	93.23%	94.12%	48.90%
shEMCN	46.02M	6.90G	93.37%	93.84%	49.00%

Sequencing coverage of different samples

Sample	Seqencing coverage
EMCN-L1	6.69G
EMCN-L2	6.78G
EMCN-L3	7.02G
EMCN-LLC-L1	6.84G
EMCN-LLC-L2	7.03G
EMCN-LLC-L3	6.45G
WT-L1	6.62G
WT-L2	6.87G
WT-L3	7.01G
WT-LLC-L1	6.57G
WT-LLC-L2	6.69G
WT-LLC-L3	6.57G
Con313	7.01G
shEMCN	6.45G
